# Growth Performance and Whole-Plant Bacterial Assemblages of *Spirodela polyrhiza* Under Swine Biogas Slurry Dilution Gradients

**DOI:** 10.3390/plants15213349

**Published:** 2026-10-29

**Authors:** Jiaquan Li, Yaoru Xie, Ya Gao, Jiazhou Li, Cheng Ran

**Affiliations:** Heyuan Branch, Guangdong Laboratory for Lingnan Modern Agriculture, Heyuan 517000, China; dtsys_lijiaquan@163.com (J.L.); yaoruxie@icloud.com (Y.X.); gy_elegance@163.com (Y.G.)

**Keywords:** *Spirodela polyrhiza*, swine biogas slurry, bioremediation, whole-plant bacterial assemblages, nutrient recycling

## Abstract

Swine biogas slurry is a nutrient-rich livestock effluent whose treatment could be coupled with plant biomass production. We evaluated the short-term bioremediation potential of *Spirodela polyrhiza* by cultivating plants for 7 days in 5–20% (*v*/*v*) slurry or reference media. We measured growth and aqueous-phase chemistry and characterized bacterial assemblages recovered from rinsed whole plants using 16 S rRNA sequencing. In the primary experiment, the 5% slurry treatment showed the highest mean fresh biomass and fresh-weight relative growth rate among slurry treatments, although differences in growth were modest. Apparent aqueous-phase NH_3_-N concentrations decreased by more than 90% across all four slurry dilutions. In a supplementary experiment using a separate slurry batch, dry-weight relative growth rates did not differ significantly among the four slurry treatments (*p* = 0.168). After volume correction, NH_3_-N depletion was 94.6–96.8% in plant-containing systems versus 20.8–27.9% in plant-free controls; corresponding total nitrogen depletion was 91.5–93.9% versus 19.7–29.0%. Slurry amendment was associated with generally higher bacterial richness, while Shannon diversity was highest at 5%; whole-plant community composition differed among treatments. Together, these short-term findings show how the growth of *S. polyrhiza*, aqueous-phase nutrient depletion, and whole-plant bacterial assemblages varied with slurry concentration. They provide a basis for further evaluation of duckweed-assisted biogas slurry treatment in relation to biomass production and nutrient recycling.

## 1. Introduction

Swine biogas slurry is the nutrient-rich liquid digestate remaining after anaerobic digestion of swine manure and typically contains ammoniacal nitrogen, phosphorus-containing nutrients, soluble salts, and residual organic matter. When these nutrient-rich effluents are discharged or applied without adequate treatment, they can lead to nutrient pollution and organic loading in the receiving environments. At the same time, the residual nutrients in these streams are recoverable resources that can be recycled through plant-based systems, an approach that aligns with circular economy principles for managing livestock manure digestate [1,2,3].

Duckweeds are small, fast-growing floating aquatic plants capable of converting dissolved nutrients into harvestable biomass. Their use in phytoremediation, nutrient removal, biomass production, and downstream bioproduct development has been extensively studied [4,5,6]. Of all the duckweed species, *Spirodela polyrhiza* is particularly suitable for slurry-based cultivation because of its rapid vegetative growth, simple morphology, and tolerance of eutrophic conditions [7,8,9,10]. However, cultivating duckweed in digestate-derived media is not merely a matter of providing adequate nutrients. High ammonium concentrations, elevated electrical conductivity, residual chemical oxygen demand, or imbalanced nutrient profiles can suppress plant performance, alter water chemistry, and disturb the biological equilibrium of the cultivation system.

Plant-associated bacterial communities play important roles in nutrient transformation, surface colonization, stress adaptation, and plant–environment interactions. In plant microbiome research, community composition is shaped by host characteristics, plant compartments, and environmental factors [11,12,13]. In floating aquatic plants, direct contact between plant surfaces and the surrounding water enables the rapid colonization and assembly of environmental microorganisms in response to changing nutrient conditions. Duckweed-associated microbiomes have thus become increasingly relevant for understanding plant performance and system stability in nutrient-rich aquatic environments. Recent studies have begun to characterize these microbial assemblages. Duckweeds harbor bacterial communities that share structural features with terrestrial leaf microbiomes, and these communities can shift dynamically after transfer from natural habitats to nutrient-deficient conditions [14,15]. A study on *Wolffia globosa* showed that duckweed-associated bacterial communities contain conserved taxa and predicted functional traits related to colonization, motility, chemotaxis, and host adaptation [16]. Work on *S. polyrhiza* further demonstrated that frond-associated bacterial communities can be restructured under cadmium stress [17], and that individual duckweed-associated bacterial isolates exert isolate-specific effects on host growth, carbon allocation, and transcriptomic responses [18].

An important question for duckweed-assisted bioremediation is whether plant growth on nutrient-rich slurry is accompanied by reduced aqueous-phase chemical burden. Biomass production and nutrient depletion need not vary in parallel: slurry dilution changes the nitrogen, phosphorus, salt, and organic matter loading experienced by plants and associated microorganisms. Bacterial assemblages recovered from duckweed provide complementary information on the biological conditions associated with treatment, although endpoint 16 S profiles cannot establish microbial contributions to depletion. Despite extensive studies on duckweed biomass production and wastewater treatment, the combined short-term responses of *S. polyrhiza* growth, residual swine slurry chemistry, and rinsed whole-plant bacterial assemblages remain insufficiently characterized. Because these chemical variables covary along a dilution gradient, concentration gradient experiments cannot isolate the effects of individual drivers [19,20].

In this study, we investigated the short-term potential of *S. polyrhiza*-assisted treatment of swine biogas slurry, using slurry concentration as an experimental variable to assess plant growth alongside changes in the aqueous-phase chemical burden. We compared biomass accumulation and photosynthetic pigment status across slurry dilutions and reference media and measured apparent changes in NH_3_-N, TN, TP, and COD in the primary plant-containing experiment. A supplementary experiment using a separately collected slurry batch included parallel plant-containing and plant-free vessels to assess background depletion and additional depletion associated with plant presence. We also characterized rinsed whole-plant bacterial assemblages and examined their associations with residual chemistry and 16 S-inferred functional potential. We hypothesized that lower slurry loading would provide a more favorable balance between plant growth and residual chemical burden, whereas higher loading would leave stronger residual chemical gradients accompanied by changes in whole-plant bacterial assemblage composition.

## 2. Results

### 2.1. Swine Biogas Slurry Dilution Established Persistent Chemical Gradients and Treatment-Specific Apparent Nutrient Depletion

The 7-day cultivation experiment is summarized in Figure 1a–c, with plant performance shown in Figure 1a,b and apparent aqueous-phase chemical decreases shown in Figure 1c. At the start of the experiment, the four slurry dilution treatments produced clear concentration-dependent increases in EC, NH_3_-N, TN, TP, and COD (Table S1). In the 5%, 10%, 15%, and 20% slurry treatments, NH_3_-N increased from 11.79 to 49.94 mg L^−1^, TN increased from 13.36 to 56.11 mg L^−1^, TP increased from 1.56 to 5.83 mg L^−1^, and COD increased from 22.10 to 52.40 mg L^−1^. These ranges confirmed that the dilution series created distinct chemical environments for *S. polyrhiza* cultivation.

After 7 days, residual EC, NH_3_-N, TN, TP, and COD differed significantly among treatments, whereas pH remained similar across all treatments (Table S1). Treatments with higher slurry loading (15% and 20%) retained significantly higher residual concentrations of NH_3_-N, TN, TP, and COD than the lower dilution treatments. Treatments with higher slurry loading therefore maintained persistent chemical gradients at the time of plant and bacterial sampling.

In the duckweed-containing systems, apparent decreases in water column analytes varied considerably with slurry concentration and chemical species. NH_3_-N reductions reached 97.29%, 94.94%, 90.89%, and 91.11% in the 5%, 10%, 15%, and 20% slurry treatments, respectively; for TN, the corresponding reductions were 82.41%, 64.07%, 48.61%, and 46.50%; for TP, 92.09%, 90.28%, 42.13%, and 51.00%; and for COD, 30.20%, 10.12%, 6.09%, and 8.28% (Figure 1c). These concentration-based reductions describe apparent changes in water column chemistry within the original duckweed-containing experiment. Because plant-free controls and residual volume measurements were not included in that run, these values should not be interpreted as direct plant uptake or as truly plant-assimilated fractions.

### 2.2. The 5% Slurry Treatment Showed the Highest Short-Term S. polyrhiza Growth Among the Tested Conditions

Plant growth after 7 days differed among cultivation substrates (Figure 1a,b; Table S2). Final fresh weight was lowest in the ultrapure water control (7.199 ± 0.134 g) and highest in the 5% slurry treatment (9.350 ± 0.433 g), followed by the 10% slurry, 20% slurry, 15% slurry, and Hoagland solution. Fresh-weight relative growth rate followed the same pattern, reaching its maximum in the 5% slurry treatment (0.0893 ± 0.0066 d^−1^) and its minimum in the control (0.0521 ± 0.0026 d^−1^). The global treatment effect was significant for final fresh weight and fresh-weight RGR (Kruskal–Wallis, *p* = 0.0109), and Dunn’s post hoc test with Benjamini–Hochberg correction confirmed that the 5% slurry treatment was significantly higher than the control. Final dry weight and dry-weight RGR also exhibited significant global treatment effects (*p* = 0.0329), although no pairwise comparison remained significant after correction.

Total chlorophyll was higher in Hoagland (0.6977 ± 0.0454 mg g^−1^ FW) and 5% slurry (0.7077 ± 0.0060 mg g^−1^ FW) than in CK (0.5604 ± 0.0261 mg g^−1^ FW). Although the 5% slurry treatment showed the highest growth values, absolute differences among the nutrient-containing treatments were modest. Treatments with higher slurry concentrations did not further improve plant growth or chlorophyll accumulation, indicating a non-linear growth response to slurry loading. By contrast, apparent aqueous-phase NH_3_-N concentration decreases exceeded 90% in all four slurry treatments (Figure 1c). Carotenoid measurements are reported in Table S2 as complementary pigment endpoints; no mechanistic interpretation is assigned to these changes in the present study.

### 2.3. Supplementary Experiment: Plant-Containing Versus Plant-Free Aqueous-Phase Depletion and Plant Growth

To complement the primary cultivation experiment and quantify background aqueous-phase depletion, a supplementary experiment was conducted using a newly collected slurry batch from the same farm. Plant-containing (plant+) and plant-free (plant−) vessels were incubated in parallel for 7 days under comparable cultivation conditions, and growth was assessed in plant+ vessels using final dry biomass and dry-weight relative growth rate (RGR). Across all six cultivation media, Hoagland showed the highest mean final dry weight (0.872 ± 0.036 g) and dry-weight RGR (0.1336 ± 0.0059 d^−1^), closely followed by the 5% slurry treatment (0.862 ± 0.021 g and 0.1321 ± 0.0034 d^−1^, respectively). Among the four slurry dilutions, the 5% treatment had the highest mean final dry weight and dry-weight RGR, followed by 15%, 10%, and 20% slurry. Neither the global six-treatment effect (Kruskal–Wallis, H = 11.035, *p* = 0.0507) nor the comparison restricted to the four slurry treatments (H = 5.051, *p* = 0.168) was significant. Thus, these growth differences were descriptive rather than evidence of a statistically established concentration optimum (Table S3). Residual medium volumes were recorded for mass-based depletion calculations; mean day-7 volume loss was 14.1–15.3% across plant+ slurry treatments and 14.5–19.2% across plant− slurry treatments (Table S3).

Because dry-weight RGR was also reported for the primary experiment (Table S2), growth rates can be compared between experiments using the same biomass basis. For the 5%, 10%, 15%, and 20% slurry treatments, respectively, mean dry-weight RGRs were 0.0916, 0.0878, 0.0790, and 0.0891 d^−1^ in the primary experiment, compared with 0.1321, 0.1233, 0.1279, and 0.1136 d^−1^ in the supplementary experiment (Table S3). The 5% treatment had the highest mean dry-weight RGR in both experiments, but the relative ordering of the other slurry treatments was not preserved. These values are compared descriptively because the experiments used independent slurry batches; the data were not pooled for statistical testing.

As illustrated in Figure 2, after volume correction, NH_3_-N depletion in plant-containing systems ranged from 94.6% to 96.8% across the 5–20% slurry treatments, compared with 20.8% to 27.9% in plant-free systems. Corresponding values for TN were 91.5% to 93.9% with plants and 19.7% to 29.0% without plants. In the absence of plants, depletion of TP and COD remained low, at 5.8–9.2% and 4.0–7.1%, respectively. By contrast, plant-containing systems achieved TP depletion of 56.8–95.2% and COD depletion of 10.3–39.2%. Compared with plant-free vessels, plant-containing vessels showed additional volume-corrected aqueous-phase depletion of 68.4–74.7 percentage points for NH_3_-N, 65.0–71.8 for TN, 50.9–86.8 for TP, and 3.3–35.2 for COD.

These results show that background aqueous-phase losses were measurable without plants, particularly for nitrogen, but remained substantially lower than the depletion observed in plant-containing systems. The additional depletion associated with plant presence was most pronounced for NH_3_-N and TN, whereas the difference for COD was smaller and more dependent on slurry concentration. Considered alongside the primary experiment, the supplementary findings show the same qualitative combination of modest differences in growth across slurry concentrations and consistently high aqueous-phase NH_3_-N depletion, although the concentration-based and mass-based percentages are not directly interchangeable. These differences should be viewed as system-level responses associated with the presence of *S. polyrhiza* rather than direct estimates of nutrient assimilation by the plant.

The Hoagland mineral nutrient reference also showed greater TP depletion in plant-containing than in plant-free vessels. Using the measured initial TP concentration (30.30 mg L^−1^) and the measured day-7 volumes, volume-corrected TP depletion was 74.43 ± 1.54% in plant-containing vessels and 7.90 ± 0.08% in plant-free vessels (Table S3). Thus, the plant-containing minus plant-free difference was 66.53 percentage points in this reference medium. These data do not identify the processes responsible for this difference, and this Hoagland-specific observation is not extrapolated to the swine-slurry treatments.

### 2.4. Slurry Concentration Was Associated with Shifts in Whole-Plant Bacterial Taxonomic Structure

Whole-plant bacterial assemblages recovered from rinsed *S. polyrhiza* biomass differed markedly among cultivation substrates (Figure 3). At the phylum level, Pseudomonadota dominated all treatments, with its highest relative abundance in the control and lower abundances under the 10% and 15% slurry treatments. Actinomycetota was considerably more abundant in the 10% and 15% slurry treatments than in the control, Hoagland solution, or 5% slurry treatment. Bacteroidota was detected in all groups, with higher proportions in Hoagland solution and low-concentration slurry treatments.

At the genus level, a large proportion of sequences belonged to low-abundance taxa classified as Others, indicating that *S. polyrhiza* harbored taxonomically complex whole-plant assemblages under the tested conditions. Several genera displayed clear concentration-related patterns. *Cellvibrio* was more abundant in slurry treatments, particularly in the 20% slurry treatment, whereas *Streptomyces* was enriched mainly in the 10% and 15% slurry treatments. The genus-level heatmap further revealed treatment-specific signatures. The 20% slurry treatment was characterized by *Stenotrophomonas*, *Acidovorax*, *Hydrogenophaga*, *Nocardioides*, *Cellvibrio*, *Rheinheimera*, *Inhella*, and *Pseudomonas*. The 10% and 15% slurry treatments were associated with *Streptomyces*, unclassified *Saprospiraceae*, *Novispirillum*, and *Niveispirillum*. In contrast, Hoagland solution was associated with higher abundances of *Aquabacterium*, *Methylophilus*, *Piscinibacter*, *Novosphingobium*, and *Flavobacterium*. These findings demonstrate that slurry concentration was associated with distinct whole-plant bacterial assemblages on *S. polyrhiza*.

### 2.5. Slurry Treatments Were Associated with Increased Bacterial Richness, but Diversity Responses Were Non-Linear

Alpha diversity analysis indicated that nutrient amendment and slurry addition expanded the detectable bacterial pool associated with *S. polyrhiza* (Figure 4). Chao1 and observed features were lowest in CK and higher in Hoagland and slurry treatments. The observed features index was significantly increased under the 20% treatment compared with CK. Shannon diversity was also lowest in CK and higher in Hoagland and slurry treatments. The 5% slurry treatment showed the highest Shannon index, followed by 20% slurry, whereas 10% and 15% slurry showed intermediate Shannon values despite relatively high richness.

The absence of a strictly linear diversity response suggests that low slurry loading may have increased taxon richness and evenness, whereas moderate or high slurry loading may have favored enrichment of particular bacterial groups. Additional Simpson, Good’s coverage, and Pielou’s evenness results are provided in Figure S1.

### 2.6. Bacterial Community Structure Separated Among Cultivation Substrates

Principal coordinate analysis based on Bray–Curtis dissimilarity separated the control, Hoagland, and slurry treatments, with the first two axes explaining 74.28% of the total variation (Figure 5a). Control samples clustered in the negative region of both PC1 and PC2, while slurry-treated samples were distributed predominantly toward the positive side of PC1. Non-metric multidimensional scaling ordination further supported treatment-dependent separation (Figure 5b), with the 5% slurry treatment separated from higher-concentration slurry treatments along NMDS2.

Global multivariate tests confirmed that bacterial community structure differed significantly among treatments (Table 1). PERMANOVA detected a strong treatment effect (*F* = 55.140, *R*^2^ = 0.958, *df* = 5, 12, *p* = 0.001), ANOSIM showed complete separation among groups (R = 1.000, *p* = 0.001), and MRPP also supported among-group differentiation (*A* = 0.741, observed *δ* = 0.1095, expected *δ* = 0.4234, *p* = 0.001). PERMDISP did not reach the conventional significance threshold (*F* = 2.030, *df* = 5, 12, *p* = 0.083), but the marginal *p* value leaves some uncertainty regarding heterogeneity of dispersions, particularly given that only three biological replicates were available per treatment. The ordination separation was therefore interpreted as strong treatment-associated differentiation, with the caveat that dispersion effects cannot be fully excluded.

### 2.7. Exploratory LEfSe Screening Identified Candidate Treatment-Associated Discriminatory Taxa

Exploratory LEfSe analysis identified 34 candidate discriminatory bacterial clades with LDA scores greater than 4.0 (Figure 6). In the control treatment, candidate taxa were mainly affiliated with Pseudomonadota, including Gammaproteobacteria, *Burkholderiales*, and *Comamonadaceae*, as well as *Rhizobiaceae* and *Ensifer*. In the Hoagland treatment, candidate taxa included Bacteroidota, *Bacteroidia*, *Methylophilaceae*, *Methylophilus*, and *Aquabacterium*.

Slurry treatments showed concentration-associated discriminatory patterns. The 5% slurry treatment was mainly characterized by Alphaproteobacteria, whereas the 10% slurry treatment was represented by Lysobacterales. The 15% slurry treatment was characterized by Actinomycetota-related candidate taxa, including Actinomycetes, Kitasatosporales, Streptomycetaceae, and *Streptomyces*. The 20% slurry treatment had the largest number of candidate discriminatory taxa, including Pseudomonadales, *Cellvibrionaceae*, *Cellvibrio*, *Acidovorax*, *Hydrogenophaga*, Rhodobacterales, *Nocardioides*, Propionibacteriales, and Sphingomonadales. Given the compositional nature of 16 S data and the limited biological replication (*n* = 3), these taxa are best considered exploratory candidates associated with particular treatments rather than validated biomarkers or confirmed differentially abundant taxa.

### 2.8. Residual Chemical Gradients Were Associated with Slurry-Responsive Bacterial Genera

Environmental association analysis linked residual medium chemistry to variation in bacterial community composition. After variance inflation factor screening, pH and chemical oxygen demand were retained in the redundancy analysis model. The first two RDA axes explained 100% of the constrained variation, with RDA1 and RDA2 accounting for 76.36% and 23.64%, respectively (Figure 7a). Control and Hoagland samples were distributed mainly on the negative side of RDA1, whereas the 10%, 15%, and 20% slurry samples were positioned toward the positive side. The 20% slurry treatment aligned closely with the COD vector, indicating that residual organic matter was strongly associated with community shifts at high slurry loading. Vector fitting confirmed that both COD and pH were significantly correlated with the ordination (Table 2).

Spearman correlation analysis further revealed genus-specific associations between dominant genera and residual chemical gradients (Figure 7b). COD was positively correlated with *Cellvibrio*, *Streptomyces*, *Acidovorax*, *Hydrogenophaga*, and unclassified *Saprospiraceae*, whereas negative correlations were observed with *Ensifer*, *Methylotenera*, and *Methylophilus*. NH_3_-N showed strong positive correlations with *Cellvibrio*, *Hydrogenophaga*, and unclassified *Saprospiraceae*, and negative correlations with *Ensifer* and *Methylophilus*. *Acidovorax* showed positive correlations with EC, TN, and TP, and a negative correlation with pH. These relationships support the interpretation that slurry-derived organic matter and nutrient gradients were linked to concentration-associated bacterial restructuring.

### 2.9. 16 S-Inferred Functional Profiles Varied Across Treatments but Require Cautious Interpretation

PICRUSt2, BugBase, and FAPROTAX analyses were used to explore whether taxonomic shifts were accompanied by changes in predicted functional potential. PICRUSt2-predicted MetaCyc pathway profiles separated among treatments, with the 20% slurry treatment clearly distinct from the other groups along PC1 and the Hoagland treatment separated along PC2 (Figure 8a). BugBase-predicted phenotypes also differed among treatments (Figure 8b). Gram-negative bacteria represented the dominant predicted phenotype across all treatments, whereas the predicted proportion of Gram-positive bacteria increased mainly in the 10% and 15% treatments, consistent with the enrichment of Actinomycetota-related taxa under those conditions.

FAPROTAX profiles displayed treatment-associated patterns (Figure 8c,d). Predicted chemoheterotrophy and aerobic chemoheterotrophy were dominant and enriched mainly in the 10%, 15%, and 20% slurry treatments. Predicted methylotrophy and methanol oxidation were higher in the Hoagland and 5% slurry treatments, whereas dark hydrogen oxidation and aromatic compound degradation were enhanced under the slurry treatments, particularly those with higher slurry concentrations. These inferred patterns were broadly consistent with the enrichment of *Streptomyces*, *Cellvibrio*, *Hydrogenophaga*, *Acidovorax*, and *Nocardioides* in slurry-treated samples.

Because these results were generated using 16 S rRNA gene-based prediction tools, they represent hypothetical functional potential rather than direct measurements of microbial metabolism, gene expression, enzymatic activity, or pathogenicity. These predictions should therefore be interpreted with appropriate caution.

## 3. Discussion

### 3.1. Plant Performance Reflected a Nutrient-Benefit–Stress-Load Trade-Off

The growth response of *S. polyrhiza* to slurry concentration was distinctly non-linear. The 5% slurry treatment supported the highest final fresh weight and fresh-weight relative growth rate while maintaining total chlorophyll at levels comparable to the Hoagland solution, whereas treatments with higher slurry concentrations did not produce proportional biomass gains (Figure 1; Table S2). Comparable patterns have been observed in duckweed systems exposed to livestock slurry or wastewater [21,22]. More importantly for evaluating short-term slurry treatment, apparent NH_3_-N concentration decreases exceeded 90% in all four primary slurry treatments despite the modest differentiation in biomass production. The supplementary experiment also showed the highest mean dry-weight RGR at 5% slurry, but differences among slurry treatments were not statistically significant and the rankings at 10%, 15%, and 20% differed between batches.

Although the 5% slurry treatment showed the highest RGR within this experiment, the absolute fresh-weight RGR values across all treatments (0.0521–0.0893 d^−1^) were lower than those reported for *S. polyrhiza* clones under standardized in vitro growth-rate assays. Ziegler et al. reported substantially higher RGRs under standardized cultivation conditions [23]. Several differences in experimental context may have contributed to the lower values observed here. ZB001 was pre-cultivated in Hoagland solution for 7 days rather than subjected to a longer standardization period, the experiment began at a relatively high initial surface coverage of approximately 80%, and the plants were gently rinsed before inoculation. These factors, together with the static batch cultivation conditions, were not independently tested and therefore cannot be assigned individual causal effects. The RGR values obtained here should consequently be interpreted primarily as relative treatment responses under the specific experimental conditions rather than as estimates of the maximal growth capacity of accession ZB001.

Dry-weight RGR was measured in both the primary experiment (Table S2) and the supplementary experiment (Table S3), allowing direct descriptive comparison of this growth metric across batches. Across the four slurry treatments, mean dry-weight RGRs ranged from 0.0790 to 0.0916 d^−1^ in the primary experiment and from 0.1136 to 0.1321 d^−1^ in the supplementary experiment. Mean dry-weight RGRs were higher at every slurry concentration in the supplementary experiment, and the 5% treatment showed the highest mean in both batches (0.0916 ± 0.0086 and 0.1321 ± 0.0034 d^−1^, respectively). However, the ranking of the 10%, 15%, and 20% treatments differed between experiments, and the slurry gradient comparison in the supplementary experiment was not statistically significant (*p* = 0.168). Because the experiments used different slurry batches, this comparison does not establish a fixed or universally reproducible growth optimum.

This pattern may reflect a balance between nutrient supply and chemical stress. Moderate slurry addition provides nitrogen, phosphorus, and other elements essential for biomass accumulation, whereas excessive ammoniacal nitrogen can inhibit duckweed growth and compromise photosynthetic pigment retention [21,24]. Ammonia toxicity is further modulated by pH, since ionized ammonium and un-ionized ammonia differ in their biological effects across pH ranges [25,26]. In this experiment, pH remained within a narrow range and did not differ among treatments, whereas NH_3_-N, EC, TN, TP, and COD varied concurrently. The observed plant response therefore cannot be assigned to ammonia, salinity, nutrient concentration, or organic matter in isolation. Additionally, Hoagland medium is nitrate-based while the slurry treatments were dominated by ammoniacal nitrogen, and nitrate, nitrite, and the full ionic composition were not independently measured. Since nitrogen form and the ammonium-to-nitrate ratio can influence duckweed growth and nitrogen assimilation [27], the differences between Hoagland and slurry treatments cannot be attributed solely to total nitrogen concentration or EC. Factorial designs that independently manipulate nitrogen form, ammoniacal loading, and ionic strength are required to disentangle these factors.

The final medium chemistry provides further context for these results. The 15% and 20% slurry treatments retained higher residual nitrogen, phosphorus, EC, and COD without producing proportionally greater biomass. The consistently high apparent ammonium decreases therefore did not correspond to proportional increases in plant growth. However, decreases in water column nutrient concentrations should not be equated with nutrient recovery by duckweed [28]. The supplementary experiment provided additional information: after volume correction, plant-free vessels showed approximately 20–28% NH_3_-N depletion and 20–29% TN depletion, compared with substantially greater nitrogen depletion in plant-containing vessels (Figure 2; Table S3). Background processes were not negligible, but plant presence was associated with considerable additional aqueous-phase depletion. Neither experiment identifies the fate of the depleted ammonium; plant assimilation, microbial transformations, and physicochemical processes remain unresolved. Because the supplementary experiment used a different slurry batch, its data cannot be used to retrospectively calculate directly plant-assimilated fractions in the primary experiment.

### 3.2. Whole-Plant Bacterial Assemblages Reflected Slurry-Derived System States

Bacterial communities recovered from rinsed whole-plant biomass differed strongly among cultivation substrates. Bray–Curtis ordinations revealed clear treatment-related separation, and PERMANOVA attributed 95.8% of community variation to treatment (Figure 5; Table 1). Although PERMDISP was not significant at the 0.05 level, its *p* value of 0.083 was close enough to the threshold that some contribution from dispersion heterogeneity cannot be excluded, particularly with only three biological replicates per treatment. These results align with evidence that duckweed-associated microbiomes respond dynamically to changes in nutrient conditions and the surrounding microbial environments [14,15,16]. Since the plants were neither axenic nor surface-sterilized, the profiles represent bacterial taxa recovered from rinsed whole plants rather than strictly defined endophytic or epiphytic communities. The direct contact between duckweed surfaces and the cultivation medium allows environmental bacteria to attach, persist, and compete for colonization sites [29,30]. The observed separation should therefore be interpreted as treatment-associated restructuring of whole-plant assemblages, not as a shift within a specifically resolved plant micro-niche.

The restructuring was concentration-dependent rather than a uniform enrichment of slurry-associated bacteria. Hoagland and low-slurry treatments were associated with *Methylophilus*, *Methylophilaceae*-related taxa, and *Aquabacterium*, whereas the 10% and 15% treatments showed greater representation of Actinomycetota and *Streptomyces*. The 20% treatment was distinguished by taxa including *Cellvibrio*, *Hydrogenophaga*, *Acidovorax*, and *Nocardioides*. Previous duckweed microbiome studies have likewise identified both recurrent host-associated taxa and substantial compositional turnover across environmental conditions [14,15,16,31]. Notably, *Acidovorax* has been isolated from duckweed cultivated in poultry-farm wastewater, supporting the compatibility of at least one high-slurry-associated genus with duckweed–wastewater environments [32]. The data in this study document concentration-associated whole-plant bacterial configurations, but the ecological roles and origins of these bacteria cannot be inferred from genus-level endpoint profiles.

Several enriched genera provide plausible ecological mechanisms for the concentration-related transition. *Streptomyces* and other actinomycetes are widely reported as plant-associated bacteria with capacities related to nutrient mobilization, secondary metabolite production, growth promotion, and stress interactions [33,34,35]. Their enrichment under moderate slurry concentrations may therefore reflect conditions favorable for actinomycete persistence, although no activity beneficial to plants was directly measured. Recent isolate-based work in *S. polyrhiza* further showed that the effects of bacteria on host growth and carbon allocation can be strongly isolate specific [18], reinforcing the need to avoid assigning plant-beneficial functions solely from genus-level 16 S profiles. At higher slurry loading, the enrichment of *Cellvibrio* is consistent with the extensive carbohydrate-active enzyme systems characterized in *Cellvibrio japonicus*, which enable the degradation of complex plant-derived polysaccharides [36,37]. Similarly, members of *Hydrogenophaga* and *Acidovorax* have been recovered from activated-sludge and denitrifying wastewater environments [38,39], supporting their ecological compatibility with nutrient- and organic-matter-rich conditions. These comparisons do not establish equivalent functions in the present system because genus-level 16 S profiles cannot resolve strain identity, functional genes, gene expression, or metabolic activity.

The origin of the slurry-responsive taxa remains unresolved. The experiment cannot distinguish direct microbial introduction from slurry versus persistence of pre-existing plant-associated bacteria, environmental colonization, or subsequent community interactions. Duckweed microbiomes can reorganize after environmental transitions or abiotic stress [14,15,17], while colonization success may depend on determinants of bacterial fitness and intermicrobial interactions on plant surfaces [29,40]. Fronds and roots can also harbor compositionally distinct communities [41], but these compartments were pooled in the present analysis. The microbiome findings should therefore not be generalized to root, frond, epiphytic, or endophytic niches individually. Source-resolved, compartment-resolved, and time-resolved sampling across multiple slurry sources will be necessary to identify taxon origins and evaluate generality.

### 3.3. Residual Chemical Conditions Covaried with Bacterial Assemblage Structure Along the Slurry Gradient

Residual medium chemistry covaried with whole-plant bacterial composition along the slurry gradient. After variance inflation factor screening, COD and pH were retained in the redundancy analysis and were significantly correlated with community ordination (Figure 7a; Table 2). COD was positively correlated with several slurry-responsive genera including *Cellvibrio*, *Streptomyces*, *Acidovorax*, and *Hydrogenophaga*, while NH_3_-N, TN, TP, and EC showed additional genus-specific associations (Figure 7b). Mantel tests performed within individual treatment groups did not detect significant associations between community dissimilarity and any single physicochemical variable; Mantel r values ranged from −1.000 to 1.000, with *p* values ranging from 0.167 to 1.000 (Figure S5). Given only three biological replicates per treatment, these exploratory within-group tests had limited permutation resolution and were interpreted descriptively. These patterns are consistent with previous studies on wastewater, in which ammonium, organic loading, pH, and related physicochemical variables covaried with bacterial community structure [19,20]. Because the slurry gradient changed these factors simultaneously, the observed associations do not identify any individual chemical driver as causally responsible.

Alpha diversity also responded non-linearly to slurry loading. Bacterial richness was generally higher in slurry-amended treatments than in the control, whereas Shannon diversity was highest in the 5% slurry treatment and did not increase further with increasing slurry concentration (Figure 4). Duckweed microbiome studies have similarly shown that environmental and nutrient changes can restructure associated bacterial communities while retaining both recurrent and environmentally responsive taxa [15,16]. The present pattern may reflect an expansion of the detectable bacterial pool at low slurry loading, followed by selective enrichment of particular taxa and reduced evenness at moderate or high concentrations. Nevertheless, richness and Shannon diversity do not identify the ecological mechanisms underlying community change. Microbial assembly can involve selection, dispersal, ecological drift, and diversification [42], and wastewater communities are often shaped jointly by niche-based and neutral processes [43]. The results therefore support concentration-associated bacterial restructuring, but not direct evidence of environmental filtering.

### 3.4. 16 S-Inferred Functional Profiles Generated Testable Ecological Hypotheses

The 16 S-based functional predictions were broadly consistent with the taxonomic and physicochemical shifts observed along the slurry gradient. Moderate and high slurry treatments showed higher predicted chemoheterotrophy and aerobic chemoheterotrophy, coinciding with greater residual COD and the enrichment of *Cellvibrio*, *Hydrogenophaga*, *Acidovorax*, and *Nocardioides*. Chemoheterotrophy-related functions have been reported as dominant predicted traits in compost and digestate bacterial communities [44]. In constructed wetlands, changes in influent substrate concentration and C/N ratio can also restructure bacterial communities and nitrogen-transformation pathways [45]. The enrichment of *Cellvibrio* further supports this interpretation because members of this genus possess diverse carbohydrate-active enzymes involved in the degradation of complex plant-derived polysaccharides [36,37]. Treatments with higher slurry concentration may therefore have shifted the whole-plant assemblage toward taxa with greater potential for utilizing organic substrates. However, because the samples used in this study were collected from rinsed whole plants rather than from bulk slurry, water, or sediment, agreement with previous wastewater studies should be regarded as a convergent response to organic loading; it does not constitute evidence that identical microbial processes occurred across these habitats.

In contrast, the stronger predicted methylotrophy and methanol oxidation in Hoagland and 5% slurry treatments coincided with higher abundances of *Methylophilus* and related *Methylophilaceae*. Members of this family are known to utilize C1 compounds in methanol-fed wastewater systems and have also been identified among conserved duckweed-associated bacterial taxa [31,46]. Collectively, these results suggest a concentration-associated transition from assemblages containing stronger methylotroph-related signals under mineral nutrient or low-slurry conditions to assemblages with greater predicted heterotrophic potential under higher organic loading. This finding extends previous work by linking slurry concentration, residual chemistry, whole-plant bacterial turnover, and inferred ecological potential within a single duckweed cultivation experiment. Nevertheless, PICRUSt2, BugBase, and FAPROTAX infer functions from phylogenetic or taxonomic information rather than directly measuring functional genes, transcription, enzyme activity, metabolite flux, or process rates [47,48,49]. Genus-level profiles also cannot resolve strain-specific metabolic or pathogenic traits, so any BugBase category labeled potentially pathogenic should not be interpreted as evidence of pathogenic strains or activity. These functional results should therefore be treated as hypothesis-generating evidence and validated through metagenomics, targeted functional-gene quantification, enzyme assays, or metabolite measurements.

### 3.5. Implications for Duckweed-Based Slurry Bioremediation and Valorization

From a bioremediation perspective, the key finding is that substantial plant-associated aqueous-phase nitrogen depletion was observed across slurry dilutions despite limited differences in plant growth. In the primary experiment, apparent NH_3_-N concentration decreases exceeded 90% in all four slurry treatments (Figure 1). The supplementary experiment further showed volume-corrected NH_3_-N mass depletion of 94.6–96.8% in plant-containing vessels, compared with 20.8–27.9% in plant-free controls (Figure 2; Table S3). Total nitrogen showed a similar contrast, whereas TP and COD responses differed more with slurry concentration. These observations indicate that biomass production alone is insufficient to characterize short-term treatment performance. Because nitrogen losses and transformations were not individually quantified, additional aqueous-phase depletion associated with plant presence cannot be attributed solely to plant uptake. The consistent descriptive growth advantage of 5% slurry warrants further testing, but the nonsignificant concentration effect in the supplementary experiment and the different rankings at higher concentrations do not support a confirmed operating optimum.

The whole-plant bacterial data provide ecological context for the chemical patterns but do not establish microbial mechanisms of nutrient depletion. Longitudinal wastewater studies have similarly linked changes in bacterial assemblages to water-quality dynamics and treatment performance [50]. Because this analysis was based on a single sampling endpoint, it cannot establish whether the observed communities contributed to treatment, persisted over time, or predicted performance. Future studies should combine time-resolved plant, water, and microbiome measurements with plant-free controls and nutrient mass balances to evaluate these relationships under repeated-feed or continuous cultivation.

### 3.6. Limitations and Future Directions

Several limitations constrain the interpretation and generalization of this study. First, the primary experiment was conducted over just 7 days under controlled batch conditions. The results therefore characterize short-term responses and cannot represent long-term succession, seasonal variation, repeated slurry feeding, repeated harvesting, or continuous cultivation. Second, the primary experiment used slurry from a single farm and one sampling batch, while the independent experiment used a newly collected second batch from the same farm. The supplementary experiment provided additional within-farm evidence of background aqueous-phase depletion patterns and showed agreement with the primary experiment regarding the highest mean growth among slurry treatments: the 5% slurry treatment again showed the highest mean final dry biomass and dry-weight RGR among the four slurry concentrations. However, these second-batch growth differences were not statistically significant, and only three biological replicates were available per treatment. The cross-batch agreement should therefore be regarded as qualitative support rather than confirmation of a reproducible concentration optimum, and it does not establish generality across farms, anaerobic digestion processes, storage conditions, seasons, or slurry sources. Third, each treatment contained only three biological replicates. This limits statistical power and precision for both plant and microbiome endpoints; microbiome differential-taxon and multivariate results should therefore be considered as exploratory. Initial biomass was standardized by fresh mass, but individual frond age was not resolved, which may also contribute to biological variability. Future studies should therefore use larger biological replication, for example, at least five independent replicates per treatment where feasible, together with more standardized starting plant cohorts.

The concentration-based apparent decreases in NH_3_-N, TN, TP, and COD observed in the primary cultivation experiment cannot be attributed specifically to duckweed uptake because contemporaneous plant-free controls, residual volume measurements, plant tissue N/P measurements, and complete nutrient mass balances were not included in that run. The supplementary experiment quantified background aqueous-phase depletion after volume correction, but because it used a newly collected slurry batch it was analyzed separately and was not used to correct the primary cultivation experiment. In both experiments, disappearance from the water column may reflect plant assimilation, microbial transformation or immobilization, ammonia volatilization, precipitation, sedimentation, adsorption, or other processes; the supplementary plant-free treatment quantifies their combined background effect but does not partition these mechanisms. Moreover, changes in slurry concentration simultaneously altered several physicochemical variables, preventing the RDA and correlation analyses from isolating the independent effects of COD, pH, ammoniacal nitrogen, nutrient loading, nitrogen form, or ionic conditions. Future factorial experiments should manipulate these drivers independently and incorporate direct plant nutrient measurements.

Microbial interpretation was further limited by the sampling strategy. Rinsed whole plants were analyzed without separating fronds from roots or distinguishing epiphytic and endophytic fractions, although these compartments can harbor distinct bacterial communities [41]. In addition, the raw slurry, initial plant microbiota, residual cultivation media, and airborne or background microbiota were not sequenced, making it impossible to determine whether the taxa originated directly from slurry, from environmental selection, from persistence of the initial plant microbiota, or from subsequent colonization. Because 16 S amplicon data are compositional, relative enrichment also does not necessarily indicate an increase in absolute cell abundance [51]. Source-resolved, compartment-resolved, time-resolved, and multi-batch sampling should therefore be combined with total bacterial quantification, internal standards, flow cytometry, or taxon-specific quantitative PCR.

Finally, PICRUSt2-, BugBase-, and FAPROTAX-based results represent predicted functional potential rather than direct evidence of gene abundance, expression, metabolic activity, plant-growth promotion, nitrogen-cycling rates, or pathogenicity. Future validation should prioritize shotgun metagenomics or targeted functional gene assays, followed by metabolite or enzyme measurements and isolate-based inoculation experiments for key candidate taxa. Within these constraints, the study provides short-term evidence relevant to evaluating *S. polyrhiza*-assisted slurry bioremediation, while leaving the mechanisms of nitrogen depletion and long-term performance unresolved.

## 4. Materials and Methods

### 4.1. Plant Material and Biogas Slurry

The duckweed species used in this study was *Spirodela polyrhiza*. The accession used in this study was originally collected from the wild in Guilin, Guangxi Province, China. It was subsequently conserved at the National Azolla Germplasm Resource Nursery, Institute of Ecology, Fujian Academy of Agricultural Sciences, Fuzhou, China, and is maintained in our laboratory under the identifier ZB001. One week before the experiment, ZB001 plants were transferred to Hoagland nutrient solution and pre-cultivated for 7 days. Visually healthy plants were then selected for inoculation. Immediately before inoculation, plants were gently rinsed three times with sterile water to remove residual pre-culture medium and loosely attached debris and to standardize carry-over among treatments. This rinsing procedure was not intended as surface sterilization and may have removed part of the loosely attached epiphytic fraction. The plants were not axenically cultured, and no surface sterilization was performed. Therefore, bacterial profiles in this study represent assemblages recovered from rinsed whole-plant *S. polyrhiza* rather than the complete native epiphytic community or a specifically resolved endophytic community.

Swine biogas slurry, defined here as the nutrient-rich liquid digestate remaining after anaerobic digestion of swine manure, was collected from a large-scale pig farm operated by Dongrui Food Group Co., Ltd., Heyuan, China. Before use, the raw slurry was subjected to preliminary sedimentation and filtration to remove large suspended particles while retaining organic matter and nitrogen- and phosphorus-containing nutrients.

### 4.2. Experimental Design and Cultivation Conditions

#### 4.2.1. Primary Cultivation Experiment

Plastic cultivation containers with dimensions of 21.7 cm × 15.7 cm × 6.4 cm were used. Before the experiment, all containers were immersed in 5% sodium hypochlorite solution for 30 min, rinsed three times with ultrapure water, and dried at 60 °C for 12 h in a forced-air drying oven (DKN812C, Yamato, Japan).

Six cultivation treatments were established: a nutrient-free ultrapure-water control (CK), a Hoagland mineral-nutrient reference treatment, and 5%, 10%, 15%, and 20% swine biogas slurry. CK consisted of 1.0 L ultrapure water, whereas the Hoagland treatment consisted of 1.0 L standard Hoagland nutrient solution [52]. The 5%, 10%, 15%, and 20% biogas slurry treatments were prepared by diluting raw biogas slurry with ultrapure water at the corresponding volume ratios. Specifically, 50, 100, 150, and 200 mL of raw slurry were mixed with 950, 900, 850, and 800 mL of ultrapure water, respectively, to obtain 1 L of cultivation medium for each treatment.

A completely randomized design was used, with three biological replicates per treatment. Each replicate consisted of 5.00 ± 0.10 g fresh *S. polyrhiza* cultivated in 1.0 L of the corresponding medium. Based on preliminary trials using the same container, this biomass covered approximately 80% of the liquid surface at day 0. This initial coverage was selected to reduce direct illumination of the exposed medium and thereby minimize algal development. No duckweed biomass was removed during the subsequent 7-day cultivation period. Cultures were maintained statically without mechanical agitation or medium replacement. The experiment was conducted for 7 days in a plant growth chamber (GDN-1000C, Ningbo Yanghui Instrument Co., Ltd., Ningbo, China). The cultivation temperature was maintained at 25 °C. The photoperiod was 16 h light/8 h dark, with a light period from 06:00 to 22:00, and the light intensity was 6000 lux.

#### 4.2.2. Parallel Plant-Containing and Plant-Free Experiment

A supplementary experiment was conducted using a newly collected batch of swine biogas slurry from the same farm primarily to compare aqueous-phase chemical changes in the presence and absence of *S. polyrhiza*, while also providing an independent supplementary assessment of plant growth in the plant-containing vessels. CK, Hoagland, and 5%, 10%, 15%, and 20% slurry media were prepared as described for the primary cultivation experiment. For each medium, plant-containing (plant+) and plant-free (plant−) treatments were established simultaneously with three independent replicates. The same container type, initial medium volume (1.0 L), temperature (25 °C), photoperiod (16 h light/8 h dark), light intensity (6000 lux), and cultivation duration (7 days) were used. Plant+ vessels received 5.00 ± 0.10 g fresh *S. polyrhiza*, whereas plant− vessels contained the corresponding cultivation medium without plants.

### 4.3. Physicochemical Measurements and Depletion Calculations

Physicochemical parameters of cultivation media were measured at day 0 and day 7. Day 0 measurements represented freshly prepared media before duckweed inoculation, whereas day 7 measurements represented residual cultivation media collected from each biological replicate after *S. polyrhiza* harvesting. pH, EC, NH_3_-N, TN, TP, and COD were measured using a water quality analyzer (GL-900, GreenCare, Shandong, China; SevenDirect SD23, METTLER TOLEDO, Changzhou, China). Concentrations of NH_3_-N, TN, TP, and COD were expressed as mg L^−1^, and EC was expressed as μS cm^−1^.

For the primary cultivation experiment, concentration-based apparent decrease rates were calculated as:

$$Change\;rate=\frac{C_{0}-C_{7}}{C_{0}}\times 100\%$$

where C_0_ and C_7_ represent the measured concentrations at day 0 and day 7, respectively. Change rates were not calculated for CK when the initial values were zero or near zero. Because residual medium volume was not recorded in this experiment, these values represent concentration-based apparent decreases rather than nutrient mass balances.

For the parallel plant-containing and plant-free experiment, residual medium volume was measured at day 7. In plant+ vessels, *S. polyrhiza* was gently removed using a fixed mesh positioned above the original cultivation container and allowed to drain for 5 min so that adhering cultivation medium returned to the vessel before volume measurement. For each solute, aqueous-phase mass was calculated as M = C × V, where C is the measured concentration (mg L^−1^) and *V* is the medium volume (L). Mass-based depletion was calculated as:

$$D=\frac{{M_{0}-M}_{7}}{M_{0}}\times 100\%$$

Depletion in plant− vessels was used to quantify background aqueous-phase depletion in the absence of *S. polyrhiza*. The difference between mean depletion in paired plant+ and plant− treatments at the same slurry concentration was reported as additional depletion associated with plant presence.

### 4.4. Plant Growth and Photosynthetic Pigment Measurements

At day 7, all *S. polyrhiza* biomass from each replicate was harvested. Fresh weight was measured after draining plants in mesh bags for 10 min. Dry weight was determined after drying fresh biomass at 85 °C for 48 h to constant weight. To determine initial dry weight, separate samples of *S. polyrhiza* were randomly collected from the same pre-cultivation population and growth conditions as the plants used for inoculation. Each sample was matched in fresh weight to the inoculated biomass (5.00 ± 0.10 g) and dried at 85 °C for 48 h to constant weight. The measured dry weights of these samples were used as the initial dry weights for calculating dry-weight relative growth rate (RGR). Fresh-weight relative growth rate was calculated as RGR = [ln(FW_7_) − ln(FW_0_)]/7, where FW_0_ and FW_7_ represent initial and final fresh weight, respectively. Dry-weight RGR was calculated using the same equation based on initial and final dry weight. The same dry-weight RGR calculation was applied to the plant-containing vessels in the supplementary experiment, using the recorded initial dry weight and replicate-specific final dry weights. Final dry weight and dry-weight RGR were analyzed separately for the two experiments; because both experiments measured dry-weight RGR, these values were also compared descriptively across slurry batches.

Photosynthetic pigments were extracted using 95% ethanol. Briefly, 0.2 g of fresh plant tissue was ground with quartz sand, calcium carbonate powder, and 95% ethanol, filtered into a 50 mL brown volumetric flask, and brought to volume with 95% ethanol. Absorbance was measured at 665, 649, and 470 nm using a spectrophotometer (ALLSHENG, Nano-500, Shanghai, China). Chlorophyll a, chlorophyll b, total chlorophyll, and carotenoids were calculated using standard equations [53] and expressed as mg g^−1^ FW for chlorophyll a, chlorophyll b, total chlorophyll, and carotenoids.

### 4.5. Sample Collection and DNA Extraction

At the end of the 7-day cultivation period, whole-plant *S. polyrhiza* biomass was gently rinsed three times with sterile water, without surface sterilization, to remove residual cultivation medium and loosely attached debris before bacterial community analysis. Fronds and roots were pooled, and epiphytic and endophytic fractions were not distinguished. Accordingly, all microbiome results refer to bacterial assemblages recovered from rinsed whole plants. Day-0 plant microbiota, raw-slurry microbiota, and residual-medium microbiota were not sequenced; therefore, the present analysis compares endpoint whole-plant assemblages among treatments. Samples ZB1–ZB3 represented CK, ZB4–ZB6 represented Hoagland, ZB7–ZB9 represented 5% biogas slurry, ZB10–ZB12 represented 10% biogas slurry, ZB13–ZB15 represented 15% biogas slurry, and ZB16–ZB18 represented 20% biogas slurry. Total DNA was extracted using a Tiangen plant genomic DNA extraction kit (TIANGEN Biotech (Beijing) Co., Ltd., Beijing, China) according to the manufacturer’s instructions. DNA concentration, purity, and integrity were assessed before sequencing.

### 4.6. PCR Amplification and 16 S rRNA Gene Sequencing

Bacterial community sequencing was performed by Novogene Co., Ltd., Beijing, China. The V4 region of the bacterial 16 S rRNA gene was amplified using primers 515F and 806R (Biometra Tone, Jena, Germany) [54,55]. Each PCR mixture contained 15 μL Phusion High-Fidelity PCR Master Mix, 0.2 μM of each primer, and 10 ng of genomic DNA template (GenStar, Suzhou, China). PCR amplification was performed with an initial denaturation at 98 °C for 1 min, followed by 30 cycles of 98 °C for 10 s, 50 °C for 30 s, and 72 °C for 30 s, with a final extension at 72 °C for 5 min. PCR products were purified, pooled in equimolar amounts, used for library construction, quantified, and sequenced on the NovaSeq 6000 platform (Illumina, Inc., San Diego, CA, USA).

### 4.7. Sequence Processing and Taxonomic Annotation

Raw sequencing data were demultiplexed according to barcode and primer sequences. After barcode and primer removal, paired-end reads were merged using FLASH v1.2.11 [56]. Residual primer sequences were trimmed using Cutadapt (v5.2) [57]. Quality filtering was performed using fastp v0.23.1 to obtain high-quality clean tags [58], and chimeric sequences were identified and removed [59]. Amplicon sequence variants (ASVs) were generated using the DADA2 (v1.32.0) module in QIIME2 (v2016.10) [60,61]. Taxonomic annotation was performed using QIIME2 against the SILVA 138.1 database [62]. The ASV table was normalized according to the minimum sequencing depth before downstream alpha- and beta-diversity analyses. For alpha- and beta-diversity analyses, the ASV table was rarefied to a common depth of 30,250 sequences per sample. Rarefaction curves based on Shannon diversity and observed features were generated for all 18 samples; the selected cutoff is indicated by a vertical dashed line in each panel of Figure S6.

### 4.8. Bacterial Community Composition and Diversity Analyses

Bacterial community composition was analyzed at the phylum and genus levels. Relative abundance bar plots were generated based on group-level mean abundance. For genus-level clustering analysis, the top 25 genera with the highest total abundance were selected, and a two-way clustered heatmap was generated. Alpha diversity was evaluated using Chao1, observed features, Shannon, Simpson, Good’s coverage, and Pielou’s evenness indices. Differences among treatments were tested using the Kruskal–Wallis rank-sum test [63], followed by Dunn’s post hoc test [64], with the Benjamini–Hochberg method [65].

Beta diversity was evaluated using Bray–Curtis and weighted UniFrac distance matrices [66,67]. Principal coordinate analysis (PCoA) and non-metric multidimensional scaling (NMDS) were performed to visualize differences in bacterial community structure among samples. Global differences among treatments were tested using PERMANOVA, ANOSIM, and MRPP based on Bray–Curtis dissimilarity [68,69]. Homogeneity of multivariate dispersion was assessed using PERMDISP/betadisper based on Bray–Curtis distance with 9999 permutations.

### 4.9. Identification of Differential Bacterial Taxa

Candidate treatment-associated discriminatory taxa were screened using LEfSe analysis [70]. Taxonomic levels from phylum to genus were included. The LDA threshold was set to 4.0 for the main exploratory analysis, and a complementary lower-threshold result was provided in the Supplementary Material. Because the analysis was based on compositional 16 S data with *n* = 3 biological replicates per treatment, LEfSe was treated strictly as exploratory screening; identified taxa were not interpreted as validated biomarkers, confirmed differentially abundant taxa, or functionally important organisms.

### 4.10. 16 S-Inferred Functional Prediction

Functional prediction was performed using PICRUSt2 [47], BugBase [48], and FAPROTAX [49]. PICRUSt2 was used to infer putative EC numbers, KEGG orthologs, and MetaCyc pathways from 16 S rRNA gene profiles. BugBase was used to infer bacterial phenotypic traits, and FAPROTAX was used to infer ecological functions associated with carbon and nitrogen cycling. Differences among treatments were evaluated using the Kruskal–Wallis rank-sum test followed by Dunn’s post hoc test with Benjamini–Hochberg correction. All outputs were treated as 16 S-based predictions of functional potential and not as direct evidence of gene abundance, gene expression, enzyme activity, microbial metabolic activity, or pathogenicity.

### 4.11. Environmental Association Analyses

Relationships between physicochemical gradients and bacterial community variation were evaluated using redundancy analysis (RDA), Mantel tests, and Spearman correlations. Environmental variables included pH, EC, NH_3_-N, TN, TP, and COD. RDA was performed at the genus level after screening environmental variables using the variance inflation factor method. For Figure S5, exploratory group-specific Mantel tests compared Bray–Curtis bacterial community dissimilarity with Euclidean distance for each individual environmental variable within each treatment [71]. Because each treatment contained only three biological replicates, these within-treatment Mantel tests had low permutation resolution and were interpreted descriptively. Spearman correlation was used to evaluate relationships between environmental factors and dominant bacterial genera.

### 4.12. Statistical Analysis

Experimental data were organized using Microsoft Excel 2024. Unless otherwise stated, differences among treatments in the primary cultivation experiment were assessed using the Kruskal–Wallis rank-sum test, followed by Dunn’s post hoc test with Benjamini–Hochberg correction where appropriate. Statistical significance was defined as adjusted *p* < 0.05. Physicochemical parameters and plant variables are presented as mean ± standard deviation (*n* = 3). The supplementary experiment was analyzed separately from the primary cultivation experiment. Final dry weight and dry-weight RGR in plant-containing vessels were presented as mean ± SD. Overall differences among the six cultivation media were assessed using the Kruskal–Wallis rank-sum test. Because the global treatment effect did not reach *p* < 0.05, no inferential pairwise differences were claimed. An exploratory Kruskal–Wallis comparison restricted to the four slurry concentrations (5%, 10%, 15%, and 20%) was additionally used to assess the slurry dilution gradient independently of CK and Hoagland reference media. Volume-corrected aqueous-phase mass depletion was summarized separately for plant-containing and plant-free vessels to estimate background depletion and additional depletion associated with plant presence. Data from the supplementary experiment were not used to retroactively adjust the primary cultivation experiment. The dry-weight RGRs from the primary and supplementary experiments were compared descriptively across independent slurry batches, without pooling the datasets for statistical testing. Given the limited replication (*n* = 3 per treatment), microbiome multivariate and discriminatory-taxon analyses were interpreted as exploratory and effect patterns were emphasized over dichotomous significance claims.

## 5. Conclusions

The two 7-day experiments indicate that cultivation of *Spirodela polyrhiza* in 5–20% swine biogas slurry can be accompanied by substantial aqueous-phase nitrogen depletion without proportional gains in plant biomass at higher slurry loading. The primary experiment showed apparent NH_3_-N concentration decreases above 90% across all four slurry treatments. In the supplementary experiment, volume-corrected NH_3_-N mass depletion was 94.6–96.8% in plant-containing vessels versus 20.8–27.9% in plant-free controls. Although the 5% dilution produced the highest mean growth among slurry treatments in both batches, the supplementary between-concentration differences were not statistically significant. These findings support the short-term potential of duckweed-assisted slurry bioremediation.

Whole-plant bacterial assemblages also differed among treatments. Moderate-concentration slurry treatments were associated with greater representation of Actinomycetota- and *Streptomyces*-related taxa, whereas the 20% treatment showed a distinct assemblage containing *Cellvibrio*, *Acidovorax*, *Hydrogenophaga*, *Nocardioides*, and other candidate discriminatory taxa. These community patterns covaried with residual chemical gradients, but the present design cannot determine whether taxa were introduced by slurry, selected from the initial plant microbiota or environment, or causally responsible for any plant or chemical response. The functional predictions remain hypothesis-generating only.

Neither the concentration-based apparent decreases in the primary experiment nor the volume-corrected comparisons between plant-containing and plant-free groups in the supplementary experiment determined the quantity of nitrogen assimilated into duckweed biomass. The contributions of plant assimilation, microbial transformation, and physicochemical processes remain unresolved. As both slurry batches came from one farm and each experiment lasted 7 days with three replicates per treatment, further work should test longer cultivation periods and more diverse slurry sources while incorporating direct plant N/P measurements and complete nutrient mass balances before recommending an operating dilution or process-scale application.

## Figures and Tables

**Figure 1:**
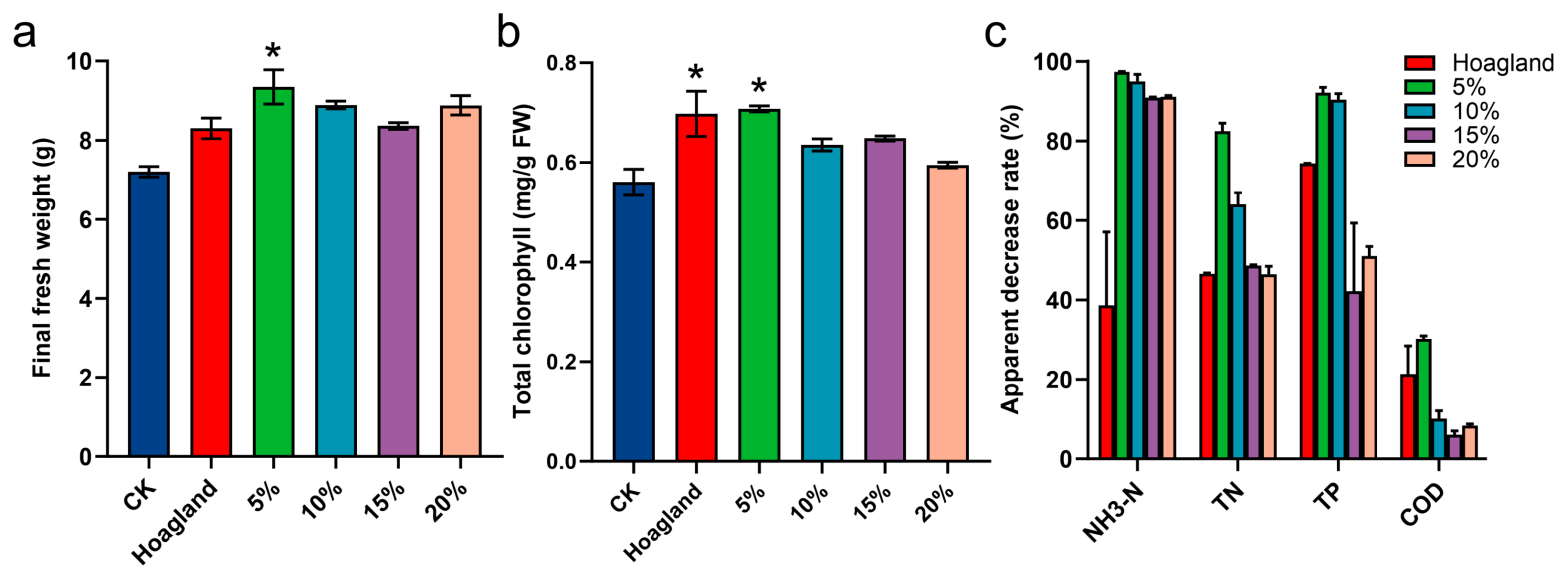
Plant performance and apparent chemical changes after 7 days. (**a**) Final fresh weight. (**b**) Total chlorophyll. (**c**) Apparent decrease rates of NH_3_-N, TN, TP, and COD. Bars show mean ± SD of three independent cultivation replicates (*n* = 3). Differences were assessed using Kruskal–Wallis tests followed by Dunn’s post hoc tests with Benjamini–Hochberg (BH) correction. * indicates *p* < 0.05 versus CK. Colors indicate treatments as shown in the legend.

**Figure 2:**
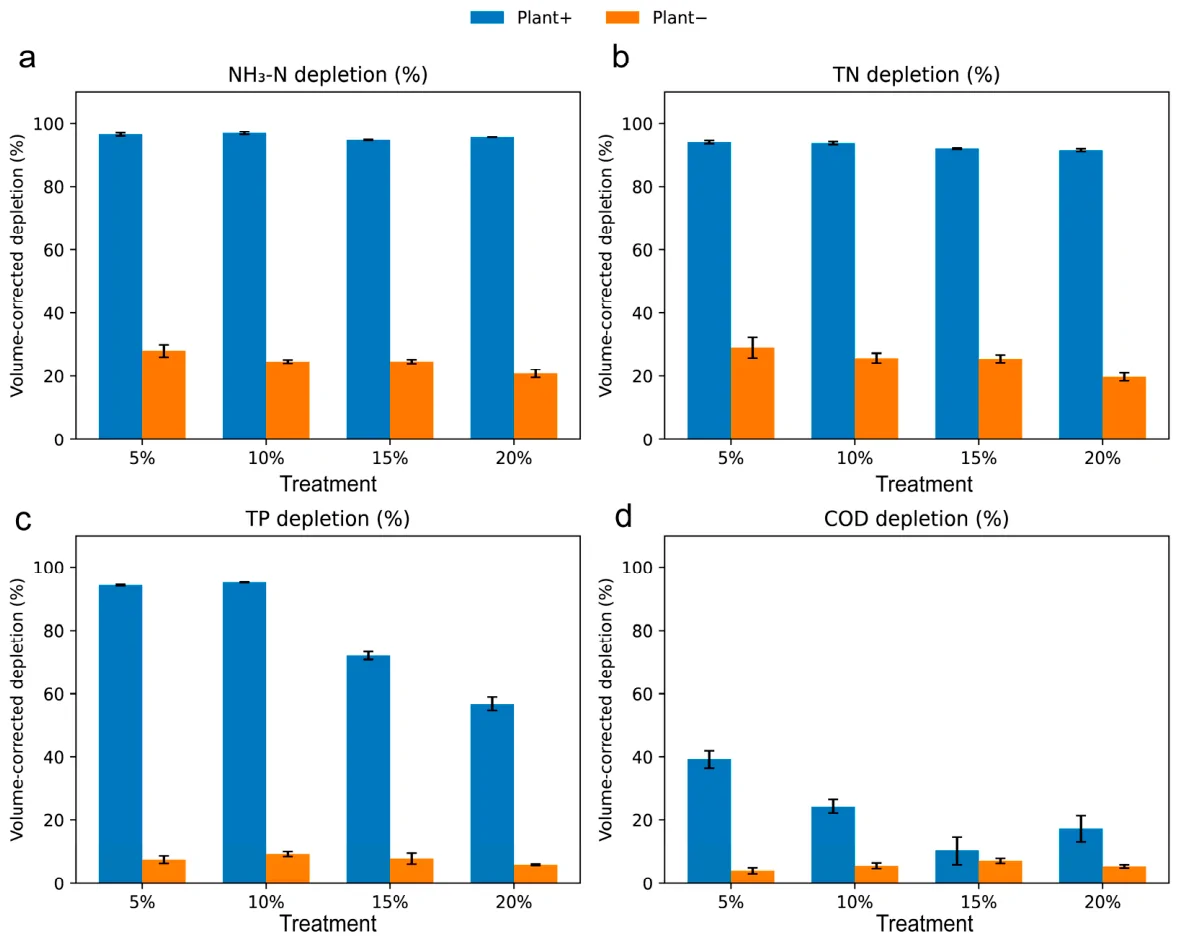
Volume-corrected aqueous-phase mass depletion in the supplementary 7-day experiment: (**a**) NH_3_-N, (**b**) total nitrogen (TN), (**c**) total phosphorus (TP), and (**d**) chemical oxygen demand (COD). Bars show mean ± SD (*n* = 3 independent vessels per treatment) for plant-containing (plant+) and plant-free (plant−) systems at 5%, 10%, 15%, and 20% (*v*/*v*) swine biogas slurry. Depletion was calculated as [(C_0_V_0_ − C_7_V_7_)/(C_0_V_0_)] × 100% using measured aqueous concentrations and volumes. Plant-free depletion reflects background aqueous-phase changes, and the additional depletion associated with plant presence is not a direct measure of plant nutrient assimilation. Exact values are listed in Table S3.

**Figure 3:**
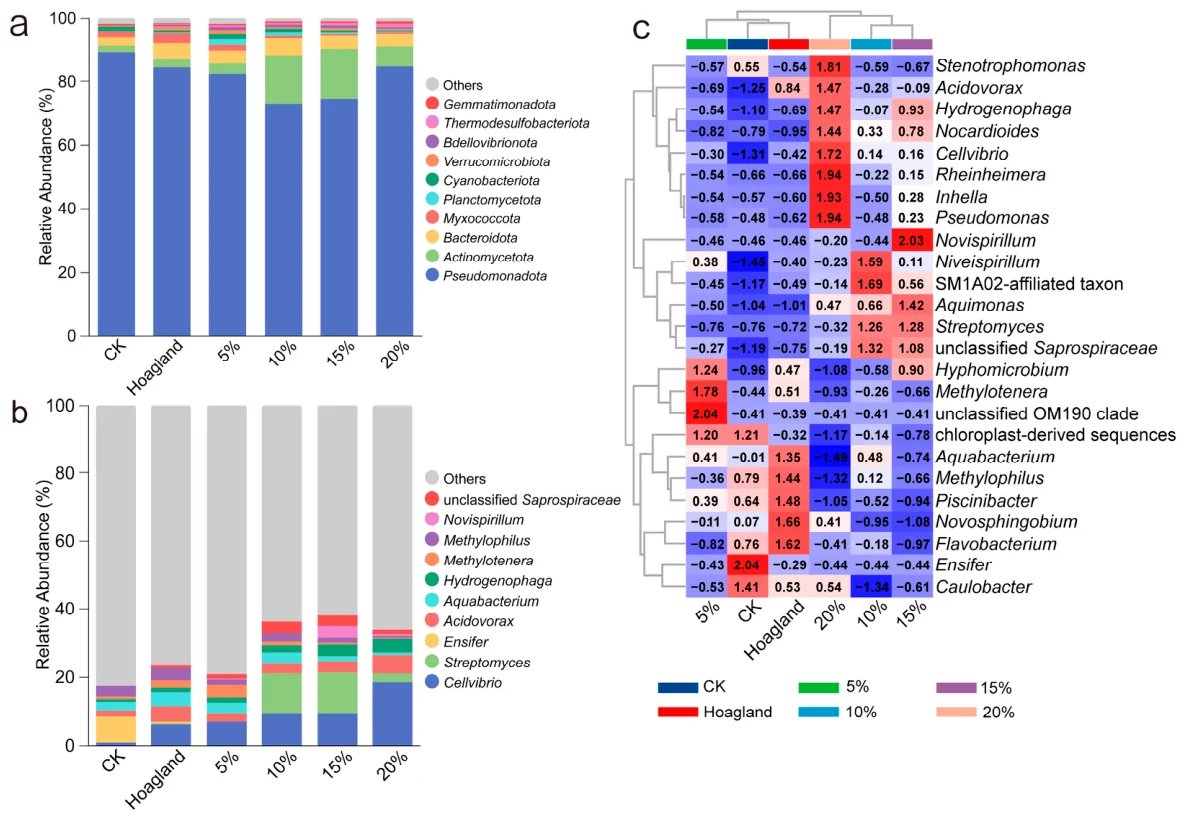
Bacterial community composition associated with *Spirodela polyrhiza*. (**a**) Relative abundance of bacterial phyla across CK, Hoagland, and slurry treatments (*n* = 3). “Others” represents the summed relative abundance of all phyla other than the 10 phyla displayed individually. (**b**) Relative abundance of dominant bacterial genera across treatments (*n* = 3). “Others” represents the summed relative abundance of all genera other than the 10 genera displayed individually. (**c**) Heatmap of the top 25 bacterial genera. Each cell shows the row-wise standardized Z-score of the treatment-level mean relative abundance. Red and blue indicate relative abundances above and below the row mean, respectively. CK, ultrapure water control; Hoagland, standard Hoagland nutrient solution; 5%, 10%, 15%, and 20%, slurry treatments at corresponding *v*/*v* concentrations.

**Figure 4:**
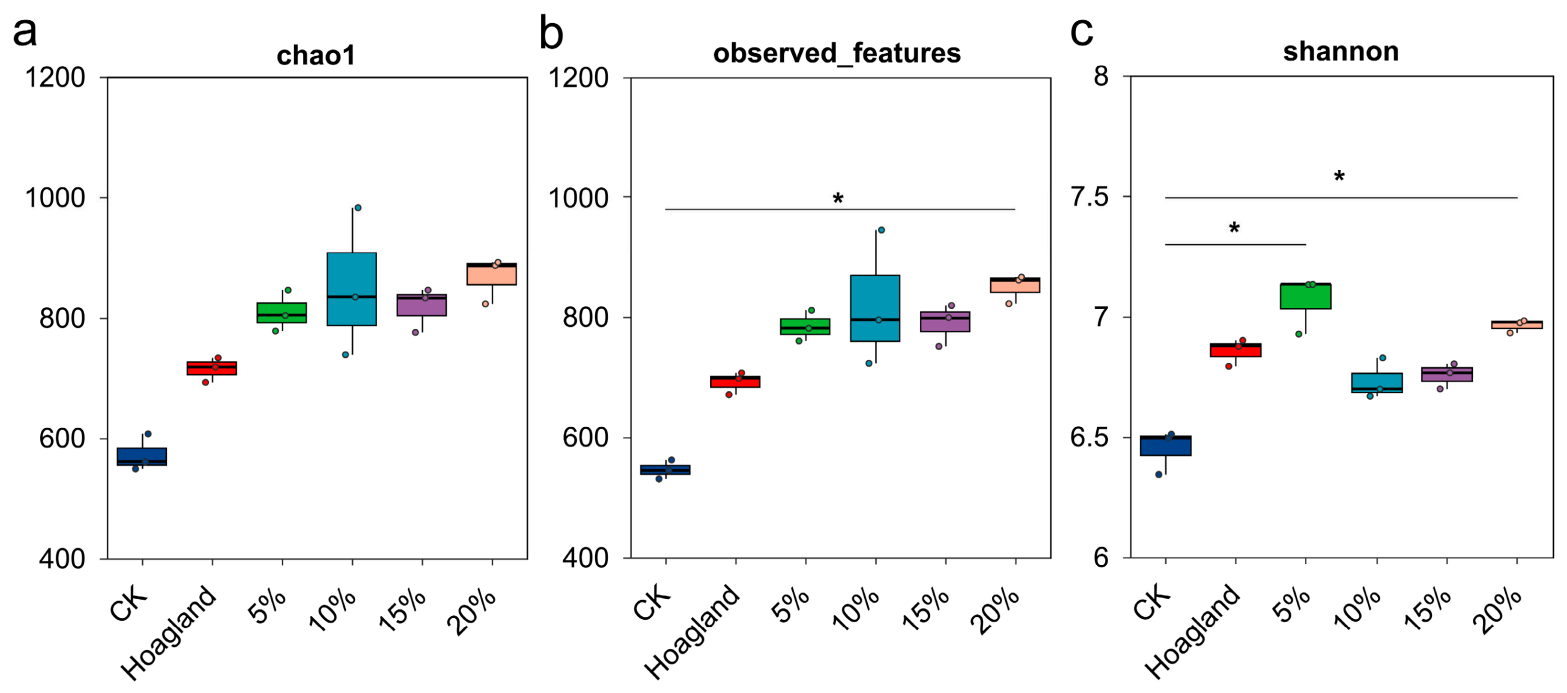
Alpha diversity of bacterial assemblages recovered from whole *Spirodela polyrhiza* biomass. (**a**) Chao1 index. (**b**) Observed features. (**c**) Shannon index. Each point represents one independent biological replicate (*n* = 3). Boxes indicate the interquartile range, central lines indicate medians, and whiskers extend to 1.5 times the interquartile range. Kruskal–Wallis tests were followed by Dunn’s tests with BH correction; * indicates *p* < 0.05 for the connected groups.

**Figure 5:**
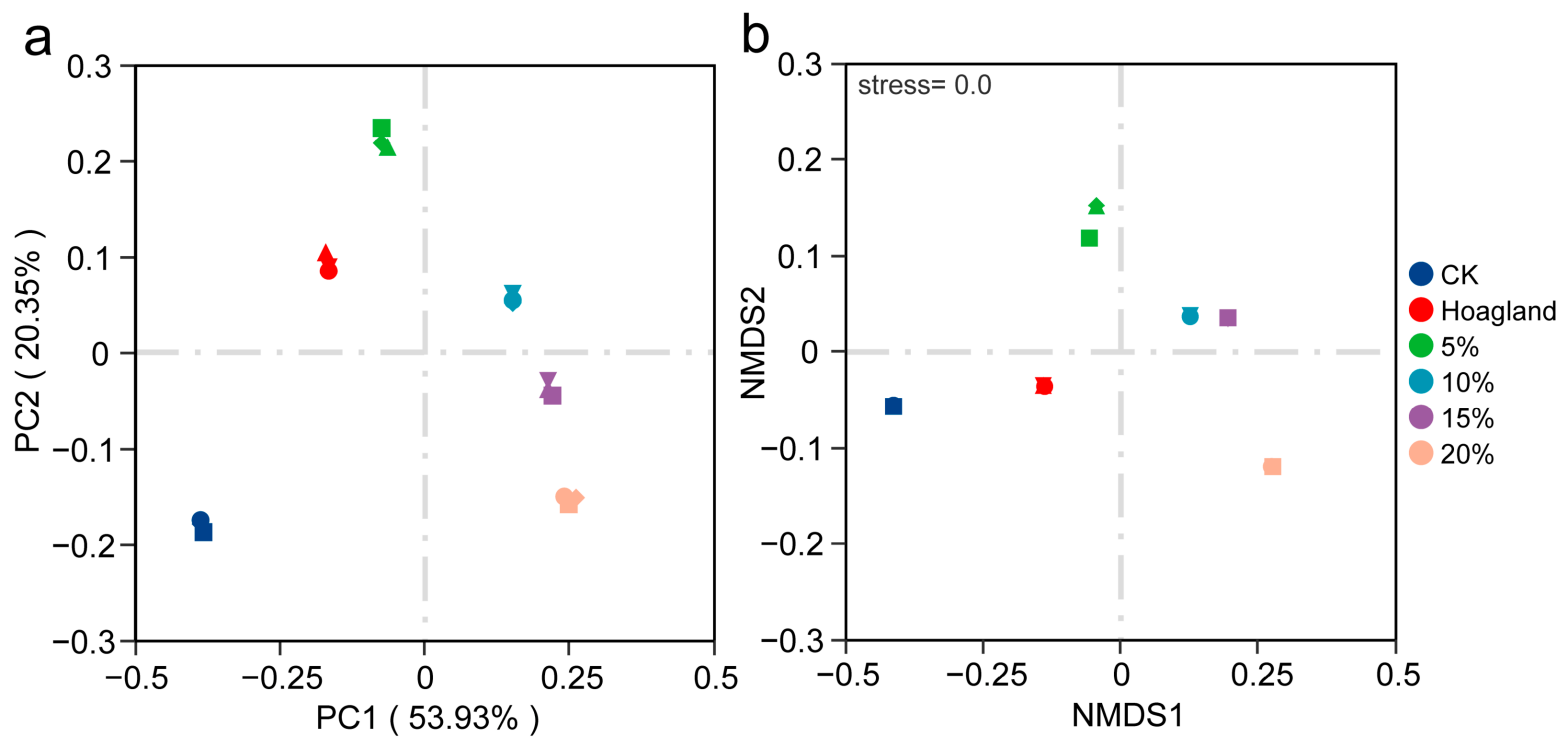
Beta diversity of bacterial assemblages associated with *Spirodela polyrhiza*. (**a**) PCoA based on Bray–Curtis dissimilarity. (**b**) NMDS based on Bray–Curtis dissimilarity. Each point represents one independent biological replicate (*n* = 3 per treatment), colors indicate treatments and the different shapes in the same treatment represent different samples. Percentages on the PCoA axes indicate the variation explained by each axis; NMDS stress is shown in panel (**b**).

**Figure 6:**
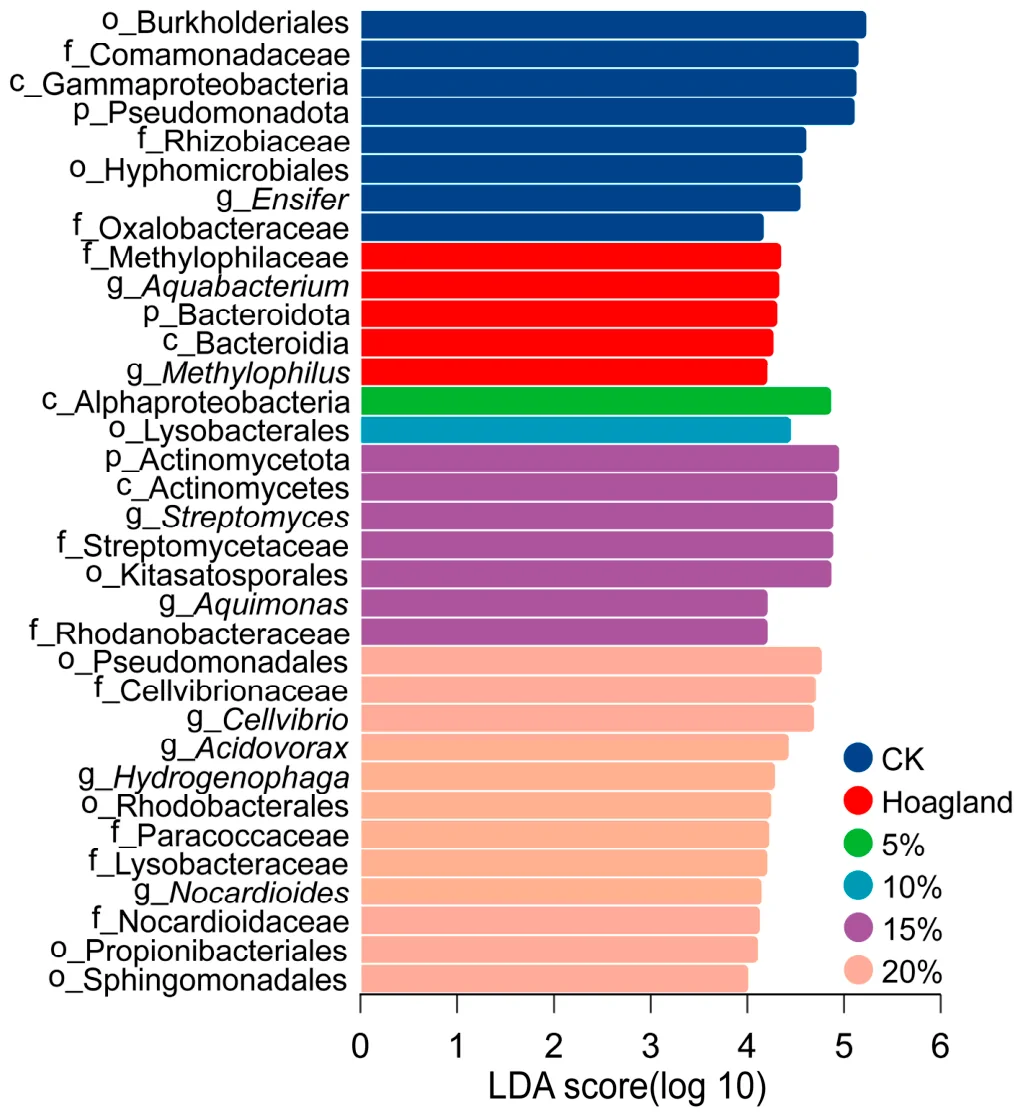
Candidate treatment-associated discriminatory taxa identified by exploratory LEfSe screening. Taxa with LDA scores > 4.0 are shown. CK, ultrapure water control; Hoagland, standard Hoagland nutrient solution; 5%, 10%, 15%, and 20%, slurry treatments.

**Figure 7:**
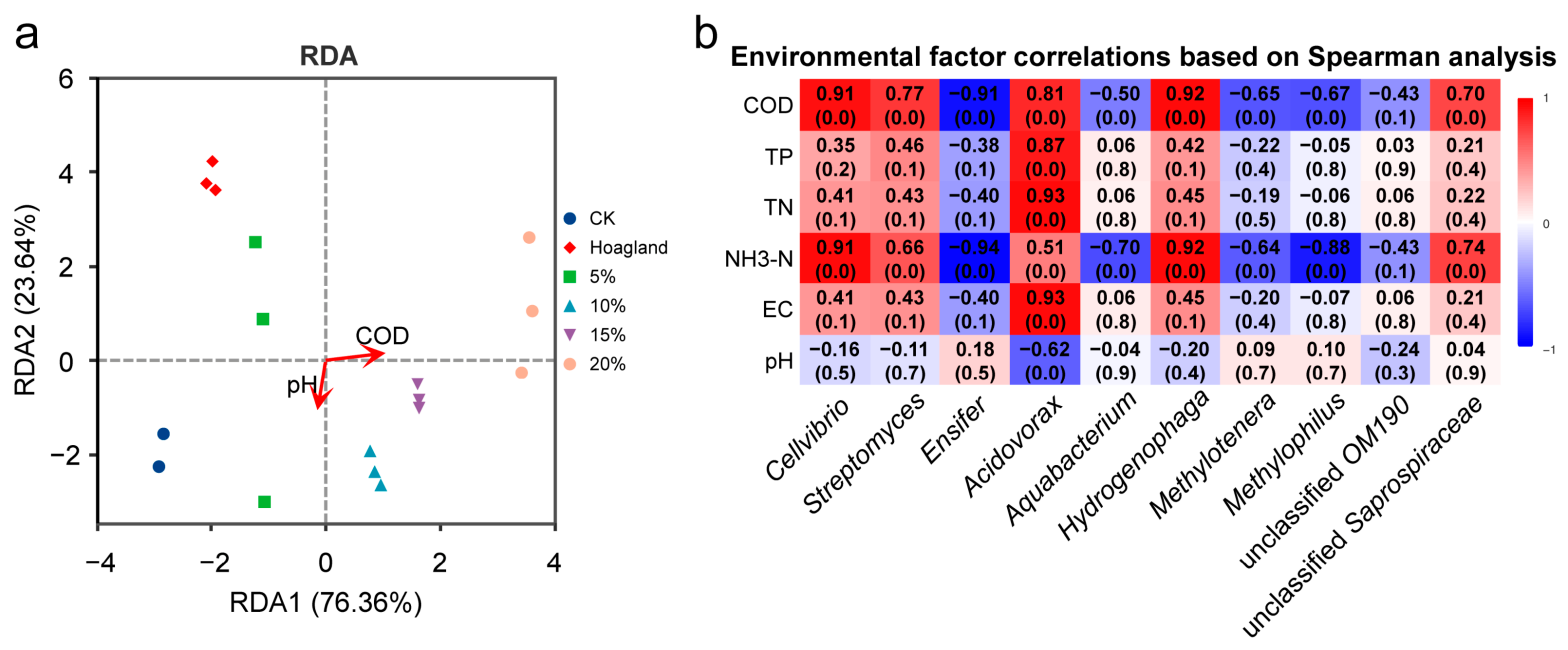
Associations between environmental variables and dominant bacterial genera. (**a**) Redundancy analysis based on genus-level community profiles and screened environmental factors. (**b**) Spearman correlation heatmap between environmental variables and dominant genera. Each point in panel (**a**) represents one independent biological replicate (*n* = 3 per treatment); arrows indicate the direction and strength of environmental associations. Panel (**b**) is a Spearman correlation heatmap, with environmental factors in rows and genera in columns. Cell values are Spearman’s r; red and blue indicate positive and negative correlations, respectively.

**Figure 8:**
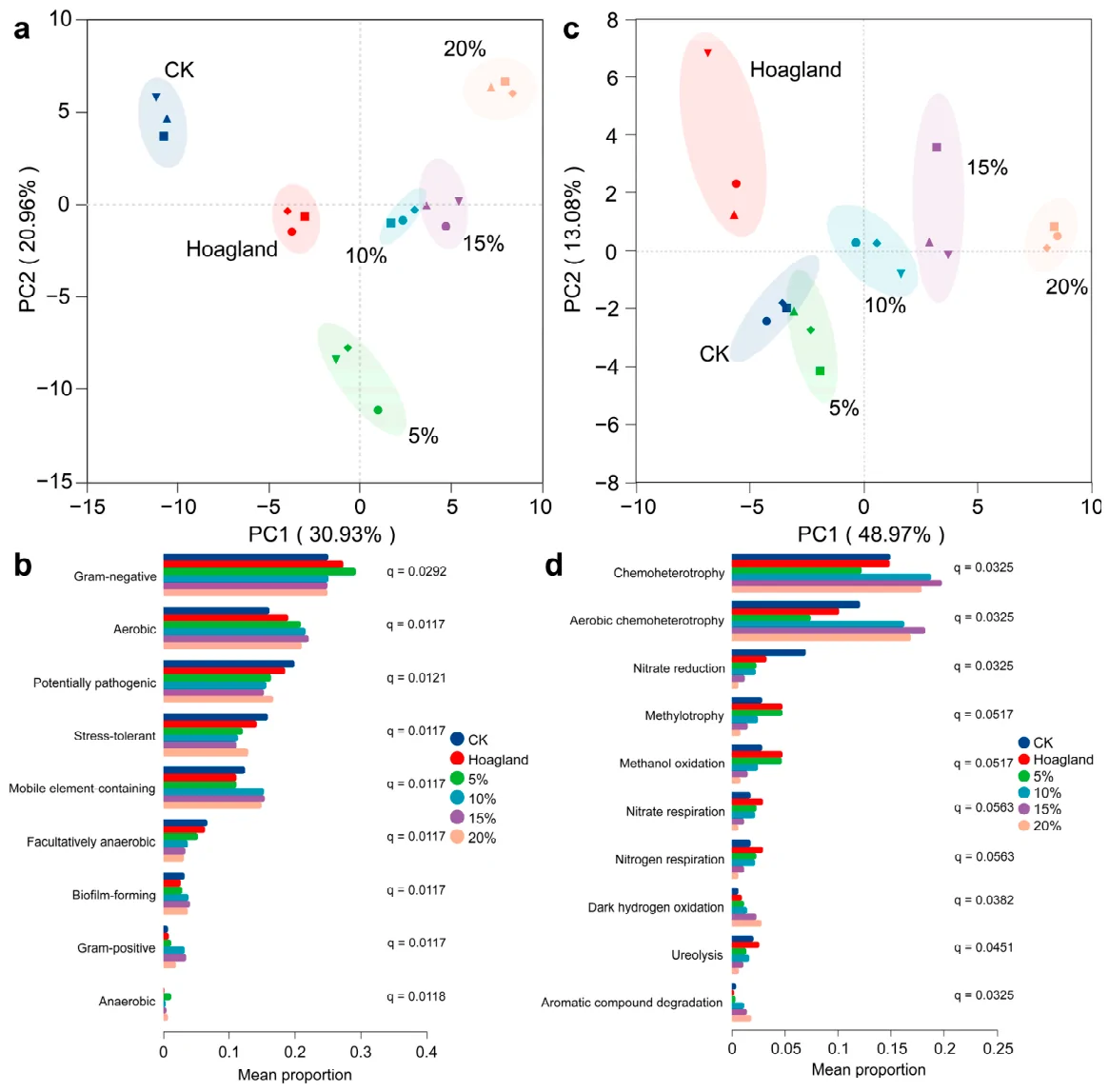
16 S-inferred functional profiles of bacterial assemblages. (**a**) PCA of PICRUSt2-predicted MetaCyc pathway profiles. (**b**) BugBase-predicted phenotypes with significant differences among treatments. (**c**) PCA of FAPROTAX-predicted ecological functional profiles. (**d**) Differential FAPROTAX-predicted ecological functions. Each point in PCA plots represents one independent biological replicate (*n* = 3), colors indicate treatments and the different shapes in the same treatment represent different samples; axis percentages indicate explained variance. Bars in panels (**b**,**d**) show mean predicted relative abundance (*n* = 3), and *q* values are BH-adjusted Kruskal–Wallis *p* values. All functions represent predictions rather than direct functional measurements.

**Table 1:** Global significance tests of bacterial community structure based on Bray–Curtis dissimilarity.

Method	Distance Metric	Comparison	Statistic	Model Information	*p* Value	Interpretation
PERMANOVA (Adonis)	Bray–Curtis	All treatments	*F* = 55.140	*R*^2^ = 0.958; *df* = 5, 12	0.001	Significant among-group difference
ANOSIM	Bray–Curtis	All treatments	*R* = 1.000	-	0.001	Complete separation among groups
MRPP	Bray–Curtis	All treatments	*A* = 0.741	Observed *δ* = 0.1095; expected *δ* = 0.4234	0.001	Significant among-group difference
PERMDISP	Bray–Curtis	All treatments	*F* = 2.030	*df* = 5, 12; 9999 permutations	0.083	Not significant at α = 0.05; dispersion effects cannot be excluded

Note: PERMANOVA, permutational multivariate analysis of variance; ANOSIM, analysis of similarities; MRPP, multi-response permutation procedure; PERMDISP, permutational analysis of multivariate dispersions.

**Table 2:** Environmental vector fitting results in RDA.

Environmental Factor	RDA1	RDA2	*r* ^2^	*p* Value
COD	0.985	0.172	0.963	0.0005
pH	−0.195	−0.981	0.856	0.0005

## Data Availability

The raw 16S rRNA gene sequencing data generated in this study are available in the NCBI Sequence Read Archive under BioProject PRJNA1468910 (https://www.ncbi.nlm.nih.gov/bioproject/PRJNA1468910/ (accessed on 10 September 2026)). The processed data supporting the findings are provided in the Supplementary Materials.

## Supplementary Materials

The following supporting information can be downloaded at https://www.mdpi.com/article/10.3390/plants15213349/s1: Table S1, Physicochemical properties of cultivation media at day 0 and day 7; Table S2, Plant growth and pigment parameters of *Spirodela polyrhiza* after 7 days of cultivation; Table S3, Plant growth and volume-corrected aqueous-phase depletion in the supplementary plant-containing and plant-free experiment, including Panel A. Growth of *S. polyrhiza* in plant-containing vessels, Panel B. Volume-corrected aqueous-phase depletion in plant-containing and plant-free systems during the supplementary experiment, Panel C. Residual medium volume loss during the supplementary experiment; Figure S1, Additional alpha-diversity indices of bacterial assemblages recovered from whole *Spirodela polyrhiza* biomass; Figure S2, Distribution of Bray–Curtis dissimilarities among bacterial assemblages across cultivation treatments; Figure S3, LEfSe-derived candidate bacterial biomarkers across cultivation treatments using an LDA threshold of 3.0; Figure S4, Supplementary predicted functional profiles of bacterial assemblages inferred from 16 S rRNA gene data; Figure S5, Mantel-test-based associations between environmental variables and bacterial community dissimilarities; Figure S6, Rarefaction analysis of the 16 S rRNA gene sequencing data for the 18 samples (ZB1–ZB18); Supplementary Data S1, sample metadata and treatment design; Supplementary Data S2, primary-experiment physicochemical parameters; Supplementary Data S3, plant growth and pigment data; Supplementary Data S4, a three-sheet workbook containing the pre-normalization ASV abundance matrix for ZB1–ZB18, taxonomic annotations with classification confidence and representative ASV sequences, and sample metadata; Supplementary Data S5, replicate-level raw measurements and volume-corrected calculations for the independent supplementary plant-containing and plant-free experiment.

## Abbreviations

The following abbreviations are used in this manuscript:

16S rRNA	16S ribosomal ribonucleic acid
ANOSIM	Analysis of Similarities
ASV	Amplicon Sequence Variant
BH	Benjamini–Hochberg
C/N	Carbon-to-Nitrogen Ratio
CK	Nutrient-free ultrapure-water control
COD	Chemical Oxygen Demand
COD/N	Chemical Oxygen Demand-to-Nitrogen Ratio
DADA2	Divisive Amplicon Denoising Algorithm 2
DNA	Deoxyribonucleic Acid
EC	Electrical Conductivity
FAPROTAX	Functional Annotation of Prokaryotic Taxa
FLASH	Fast Length Adjustment of Short Reads
FW	Fresh Weight
KEGG	Kyoto Encyclopedia of Genes and Genomes
LDA	Linear Discriminant Analysis
LEfSe	Linear Discriminant Analysis Effect Size
MRPP	Multi-Response Permutation Procedure
NCBI	National Center for Biotechnology Information
NH_3_-N	Ammonia Nitrogen
NMDS	Non-metric Multidimensional Scaling
PCA	Principal Component Analysis
PCoA	Principal Coordinates Analysis
PCR	Polymerase Chain Reaction
PERMANOVA	Permutational Multivariate Analysis of Variance
PERMDISP	Permutational Analysis of Multivariate Dispersions
PICRUSt2	Phylogenetic Investigation of Communities by Reconstruction of Unobserved States 2
QIIME2	Quantitative Insights Into Microbial Ecology 2
RDA	Redundancy Analysis
RGR	Relative Growth Rate
SD	Standard Deviation
SILVA	SILVA Ribosomal RNA Gene Database
SRA	Sequence Read Archive
TN	Total Nitrogen
TP	Total Phosphorus
VIF	Variance Inflation Factor
*v*/*v*	Volume per Volume
