# Supplementary material for: Growth Performance and Whole-Plant Bacterial Assemblages of Spirodela polyrhiza Under Swine Biogas Slurry Dilution Gradients
Source: Plants (Basel). 2026 Oct 29;15(21):3349. doi: 10.3390/plants15213349 (PMC13765669; doi:10.3390/plants15213349)
Supplement: Supplementary file 1 [file plants-15-03349-s001.zip › Supplementary Materials.pdf]

Growth performance and whole-plant bacterial assemblages of *Spirodela polyrhiza* under swine biogas slurry dilution gradients

Jiaquan Li [1], Yaoru Xie [1], Ya Gao [1], Jiazhou Li [1*], Cheng Ran [1*]

Table S1. Physicochemical properties of cultivation media at day 0 and day 7.

Treatment	Time point	pH	EC (μS cm^{-1})	NH_3-N (mg L^{-1})	TN (mg L^{-1})	TP (mg L^{-1})	COD (mg L^{-1})
CK	Day 0	6.68 ± 0.00	0.65 ± 0.00	0.00 ± 0.00	0.00 ± 0.00	0.00 ± 0.00	0.00 ± 0.00
CK	Day 7	7.30 ± 0.07	4.58 ± 1.87	0.12 ± 0.02	0.56 ± 0.09	0.10 ± 0.02	0.49 ± 0.38
Hoagland	Day 0	4.60 ± 0.00	1130.00 ± 0.00	0.19 ± 0.00	105.64 ± 0.00	30.95 ± 0.00	23.77 ± 0.00
Hoagland	Day 7	6.73 ± 0.02	1054.33 ± 19.50	0.12 ± 0.04	56.40 ± 0.15	7.94 ± 0.02	18.70 ± 1.69
5% slurry	Day 0	6.52 ± 0.00	197.30 ± 0.00	11.79 ± 0.00	13.36 ± 0.00	1.56 ± 0.00	22.10 ± 0.00
5% slurry	Day 7	7.03 ± 0.47	30.23 ± 7.55	0.32 ± 0.02	2.35 ± 0.28	0.12 ± 0.02	15.43 ± 0.17
10% slurry	Day 0	6.99 ± 0.00	386.00 ± 0.00	25.23 ± 0.00	28.14 ± 0.00	3.12 ± 0.00	31.50 ± 0.00
10% slurry	Day 7	7.31 ± 0.08	145.43 ± 11.60	1.28 ± 0.45	10.11 ± 0.80	0.30 ± 0.05	28.31 ± 0.62
15% slurry	Day 0	7.33 ± 0.00	569.00 ± 0.00	38.71 ± 0.00	43.28 ± 0.00	4.47 ± 0.00	42.60 ± 0.00
15% slurry	Day 7	7.00 ± 0.02	299.00 ± 12.29	3.53 ± 0.06	22.24 ± 0.10	2.59 ± 0.77	40.01 ± 0.40
20% slurry	Day 0	7.50 ± 0.00	753.00 ± 0.00	49.94 ± 0.00	56.11 ± 0.00	5.83 ± 0.00	52.40 ± 0.00
20% slurry	Day 7	6.88 ± 0.32	404.67 ± 12.34	4.44 ± 0.14	30.02 ± 1.11	2.86 ± 0.14	48.06 ± 0.25
Kruskal–Wallis P, Day 7	/	0.078700	0.005400	0.006500	0.005400	0.007100	0.005400

Note: Values are mean ± SD, n = 3. Day 0 values represent freshly prepared cultivation media before duckweed inoculation. Day 7 values represent residual cultivation media after 7 days of *Spirodela polyrhiza* cultivation. Statistical differences among treatments at day 7 were tested using the Kruskal–Wallis rank-sum test.

Table S2. Plant growth and pigment parameters of *Spirodela polyrhiza* after 7 days of cultivation.

Treatment	Final fresh weight (g)	Fresh-weight RGR (d^{-1})	Final dry weight (g)	Dry-weight RGR (d^{-1})	Chl a (mg g^{-1} FW)	Chl b (mg g^{-1} FW)	Total Chl (mg g^{-1} FW)	Carotenoids (mg g^{-1} FW)
CK	7.199 ± 0.134	0.0521 ± 0.0026	0.608 ± 0.019	0.0765 ± 0.0045	0.4035 ± 0.0204	0.1569 ± 0.0060	0.5604 ± 0.0261	0.0792 ± 0.0029
Hoagland	8.299 ± 0.262	0.0723 ± 0.0045	0.633 ± 0.024	0.0822 ± 0.0054	0.5122 ± 0.0352	0.1855 ± 0.0103	0.6977 ± 0.0454	0.1025 ± 0.0053
5% slurry	9.350 ± 0.433	0.0893 ± 0.0066	0.677 ± 0.040	0.0916 ± 0.0086	0.5035 ± 0.0103	0.2042 ± 0.0053	0.7077 ± 0.0060	0.1003 ± 0.0042
10% slurry	8.892 ± 0.098	0.0822 ± 0.0016	0.659 ± 0.026	0.0878 ± 0.0057	0.4558 ± 0.0134	0.1798 ± 0.0013	0.6356 ± 0.0121	0.0945 ± 0.0028
15% slurry	8.360 ± 0.087	0.0734 ± 0.0015	0.619 ± 0.005	0.0790 ± 0.0011	0.4704 ± 0.0029	0.1781 ± 0.0022	0.6485 ± 0.0051	0.0958 ± 0.0061
20% slurry	8.881 ± 0.244	0.0820 ± 0.0040	0.665 ± 0.023	0.0891 ± 0.0050	0.4295 ± 0.0059	0.1656 ± 0.0012	0.5951 ± 0.0058	0.0906 ± 0.0039
Kruskal–Wallis P	0.01	0.01	0.03	0.03	0.01	0.01	0.01	0.03

Note: Values are mean ± SD, n = 3. RGR was calculated as $\ln(W_7)–\ln(W_0)$ / 7. Pigment values were summarized from the pigment calculation sheet. All pigment concentrations (Chl a, Chl b, total chlorophyll and carotenoids) are expressed as mg g^{-1} fresh weight (FW).

Table S3. Plant growth and volume-corrected aqueous-phase depletion in the supplementary plant-containing and plant-free experiment

Panel A. Growth of *S. polyrhiza* in plant-containing vessels

Treatment	Final dry weight (g)	Dry-weight RGR (d^{-1})
CK	0.741 ± 0.027	0.1103 ± 0.0051
Hoagland	0.872 ± 0.036	0.1336 ± 0.0059
5% slurry	0.862 ± 0.021	0.1321 ± 0.0034
10% slurry	0.811 ± 0.043	0.1233 ± 0.0075
15% slurry	0.840 ± 0.078	0.1279 ± 0.0130
20% slurry	0.758 ± 0.035	0.1136 ± 0.0065

Note: Values are means ± SD ($n = 3$). Dry-weight relative growth rate was calculated as RGR = $[\ln(DW_7) - \ln(DW_0)]/7$. The overall six-treatment effect did not reach the conventional significance threshold (Kruskal–Wallis, $H = 11.035$, $p = 0.0507$). An exploratory comparison restricted to the four slurry dilution treatments was also non-significant ($H = 5.051$, $p = 0.168$). Among the four slurry concentrations, 5% slurry showed the highest mean final dry weight and dry-weight RGR. No pairwise statistical differences are claimed.

Panel B. Volume-corrected aqueous-phase depletion in plant-containing and plant-free systems during the supplementary experiment

Parameter	Treatment	Plant-containing depletion (%)	Plant-free depletion (%)	Additional depletion associated with plant presence (percentage points)
NH_3-N	CK	NA	NA	NA
	Hoagland	−229.90 ± 57.37	36.27 ± 2.23	−266.17
	5% slurry	96.36 ± 0.50	27.93 ± 1.86	68.43
	10% slurry	96.82 ± 0.44	24.64 ± 0.54	72.18
	15% slurry	94.65 ± 0.11	24.67 ± 0.62	69.98
	20% slurry	95.49 ± 0.01	20.80 ± 1.28	74.68
TN	CK	NA	NA	NA
	Hoagland	45.09 ± 1.43	23.17 ± 1.33	21.93
	5% slurry	93.93 ± 0.52	28.97 ± 3.18	64.96
	10% slurry	93.63 ± 0.45	25.75 ± 1.50	67.88
	15% slurry	91.95 ± 0.12	25.53 ± 1.23	66.42
	20% slurry	91.52 ± 0.39	19.73 ± 1.23	71.79
TP	CK	NA	NA	NA
	Hoagland	74.43 ± 1.54	7.90 ± 0.08	66.53
	5% slurry	94.29 ± 0.24	7.45 ± 1.17	86.84
	10% slurry	95.15 ± 0.11	9.21 ± 0.76	85.94
	15% slurry	72.04 ± 1.20	7.77 ± 1.73	64.27
	20% slurry	56.79 ± 2.25	5.85 ± 0.23	50.94
COD	CK	NA	NA	NA
	Hoagland	26.16 ± 2.93	1.59 ± 0.33	24.57
	5% slurry	39.19 ± 2.63	3.99 ± 0.92	35.20
	10% slurry	24.41 ± 2.28	5.54 ± 0.88	18.87
	15% slurry	10.32 ± 4.44	7.07 ± 0.79	3.25
	20% slurry	17.30 ± 4.02	5.34 ± 0.54	11.96

Panel C. Residual medium volume loss during the supplementary experiment

Treatment	Plant-containing volume loss (%)	Plant-free volume loss (%)
CK	14.10 ± 3.44	15.33 ± 1.89
Hoagland	16.50 ± 4.88	16.17 ± 2.84
5% slurry	15.17 ± 5.01	15.17 ± 4.31
10% slurry	14.43 ± 3.27	14.50 ± 2.29
15% slurry	14.07 ± 5.18	19.17 ± 2.57
20% slurry	15.33 ± 4.51	15.17 ± 2.57

Note: Values are means ± SD of three independent replicates (n = 3). Aqueous-phase mass was calculated as $M = C \times V$ and mass-based depletion as $D = (M_0 - M_7) / M_0 \times 100\%$. CK depletion percentages were not calculated because the initial concentrations of NH_3-N, TN, TP, and COD were zero. Negative depletion values indicate a net increase in aqueous-phase mass during cultivation. Plant-free depletion represents combined background aqueous-phase change in the absence of *Spirodela polyrhiza*. "Additional depletion associated with plant presence" was calculated as the difference between the mean plant-containing and plant-free depletion at the same treatment. This difference should not be interpreted as direct plant assimilation. The individual-vessel measurements and calculations underlying Table S3 are provided in Supplementary Data 5.

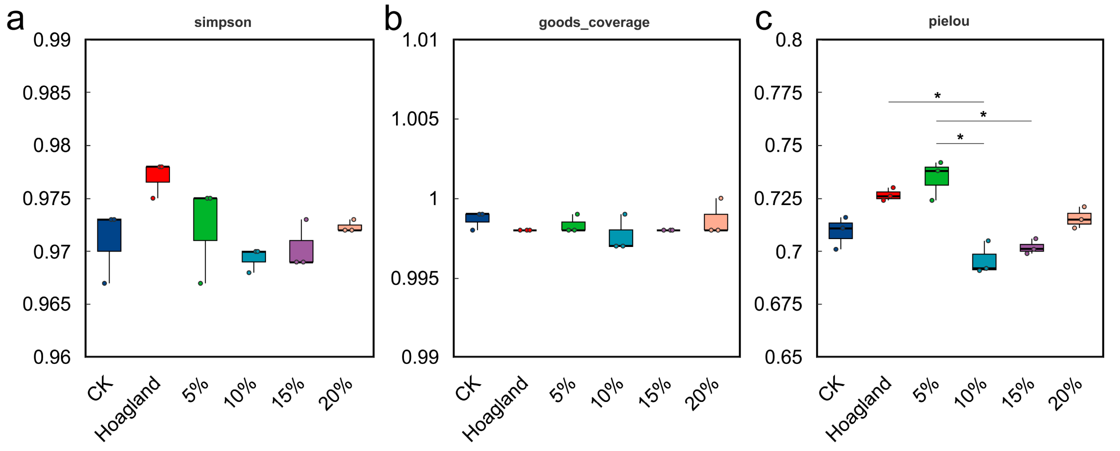

Figure S1. Additional alpha-diversity indices of bacterial assemblages recovered from whole *Spirodela polyrhiza* biomass.

Note: Boxplots showing additional alpha-diversity metrics of bacterial assemblages recovered from whole *Spirodela polyrhiza* biomass under different cultivation treatments. (a) Simpson index. (b) Good's coverage. (c) Pielou's evenness. CK, ultrapure water control; Hoagland, standard Hoagland nutrient solution; 5%, 10%, 15%, and 20%, swine biogas slurry treatments at corresponding v/v concentrations. Asterisks indicate significant differences among treatments based on Dunn's post hoc test after Kruskal–Wallis analysis with Benjamini–Hochberg correction.

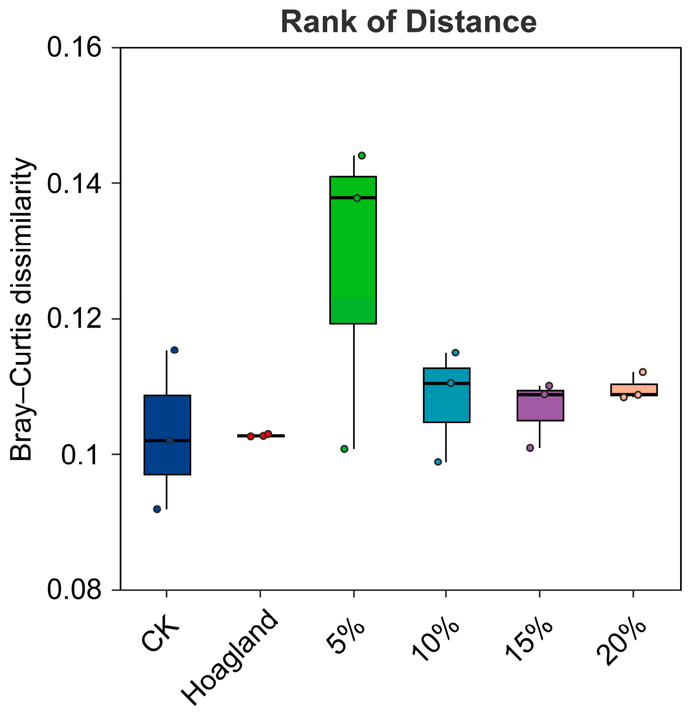

Figure S2. Distribution of Bray–Curtis dissimilarities among bacterial assemblages across cultivation treatments.

Note: Boxplot showing the distribution of Bray–Curtis dissimilarities among samples within each treatment group. CK, ultrapure water control; Hoagland, standard Hoagland nutrient solution; 5%, 10%,

15%, and 20%, swine biogas slurry treatments at corresponding v/v concentrations. The analysis was used as a supplementary beta-diversity comparison to evaluate treatment-associated differences in bacterial community dissimilarity.

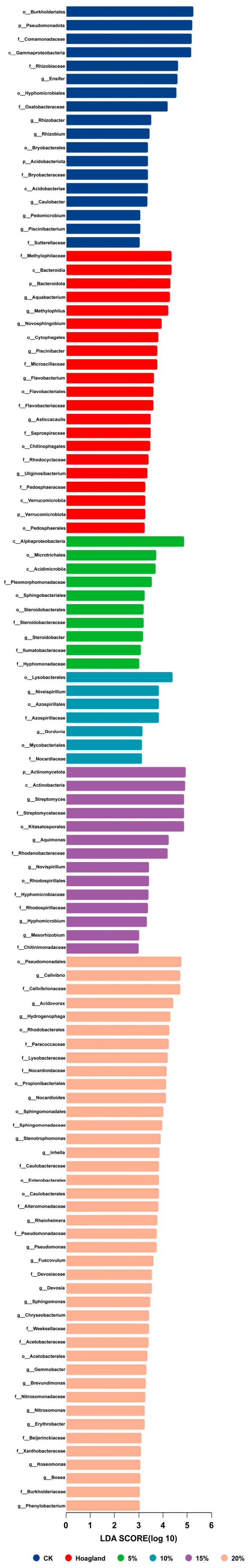
o__Burkholderiales
p__Pseudomonadota
f__Comamonadaceae
c__Gammaproteobacteria
f__Rhizobiaceae
g__Ensifer
o__Hyphomicrobiales
f__Oxalobacteraceae
g__Rhizobacter
g__Rhizobium
o__Bryobacterales
p__Acidobacteriota
f__Bryobacteraceae
c__Acidobacteriae
g__Caulobacter
g__Pedomicrobium
g__Piscinibacterium
f__Sutterellaceae
f__Methylophilaceae
c__Bacteroidia
p__Bacteroidota
g__Aquabacterium
g__Methylophilus
g__Novosphingobium
o__Cytophagales
g__Piscinibacter
f__Microscillaceae
g__Flavobacterium
o__Flavobacteriales
f__Flavobacteriaceae
g__Asticcacaulis
f__Saprospiraceae
o__Chitinophagales
f__Rhodocyclaceae
g__Uliginosibacterium
f__Pedosphaeraceae
c__Verrucomicrobiia
p__Verrucomicrobiota
o__Pedosphaerales
c__Alphaproteobacteria
o__Microtrichales
c__Acidimicrobiia
f__Pleomorphomonadaceae
o__Sphingobacteriales
o__Steroidobacterales
f__Steroidobacteraceae
g__Steroidobacter
f__Ilumatobacteraceae
f__Hyphomonadaceae
o__Lysobacterales
g__Niveispirillum
o__Azospirillales
f__Azospirillaceae
g__Gordonia
o__Mycobacteriales
f__Nocardiaceae
p__Actinomycetota
c__Actinobacteria
g__Streptomyces
f__Streptomycetaceae
o__Kitasatosporales
g__Aquimonas
f__Rhodanobacteraceae
g__Novispirillum
o__Rhodospirillales
f__Hyphomicrobiaceae
f__Rhodospirillaceae
g__Hyphomicrobium
g__Mesorhizobium
f__Chitinimonadaceae
o__Pseudomonadales
g__Cellvibrio
f__Cellvibrionaceae
g__Acidovorax
g__Hydrogenophaga
o__Rhodobacterales
f__Paracoccaceae
f__Lysobacteraceae
f__Nocardioidaceae
o__Propionibacteriales
g__Nocardioides
o__Sphingomonadales
f__Sphingomonadaceae
g__Stenotrophomonas
g__Inhella
f__Caulobacteraceae
o__Enterobacterales
o__Caulobacterales
f__Alteromonadaceae
g__Rheinheimera
f__Pseudomonadaceae
g__Pseudomonas
g__Fuscovulum
f__Devosiaceae
g__Devosia
g__Sphingomonas
g__Chryseobacterium
f__Weeksellaceae
f__Acetobacteraceae
o__Acetobacterales
g__Gemmobacter
g__Brevundimonas
f__Nitrosomonadaceae
g__Nitrosomonas
g__Erythrobacter
f__Beijerinckiaceae
f__Xanthobacteraceae
g__Roseomonas
g__Bosea
f__Burkholderiaceae
g__Phenylobacterium
0 1 2 3 4 5 6
LDA SCORE(log 10)
CK
Hoagland
5%
10%
15%
20%

Figure S3. LEfSe-derived candidate bacterial biomarkers across cultivation treatments using an LDA threshold of 3.0.

Note: Linear discriminant analysis effect size (LEfSe) showing candidate bacterial biomarkers that differed among CK, Hoagland, and swine biogas slurry treatments. Taxa with LDA scores > 3.0 are shown. Colors indicate the treatment group in which each taxon was enriched. CK, ultrapure water control; Hoagland, standard Hoagland nutrient solution; 5%, 10%, 15%, and 20%, swine biogas slurry treatments at corresponding v/v concentrations. Because LEfSe was used as an exploratory biomarker-screening approach, the identified taxa should be interpreted as candidate treatment-associated biomarkers rather than confirmed differentially abundant taxa.

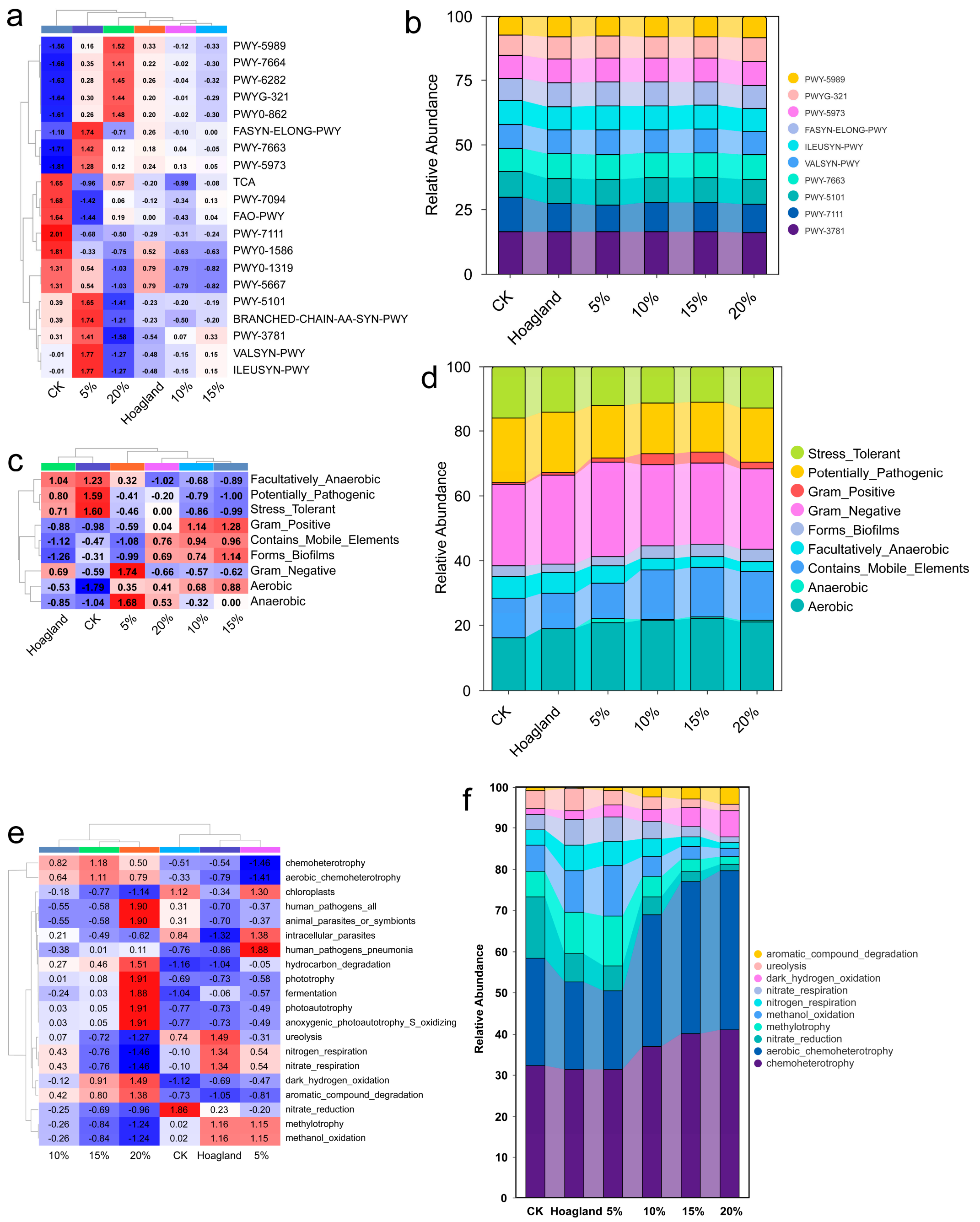

Figure S4. Supplementary predicted functional profiles of bacterial assemblages inferred from 16S rRNA gene data.

Note: Supplementary functional prediction profiles of bacterial assemblages recovered from whole *Spirodela polyrhiza* biomass under different cultivation treatments. (a) Heatmap of PICRUSt2-predicted pathway relative abundances. (b) Stacked bar plot of PICRUSt2-predicted pathway relative abundances. (c) Heatmap of BugBase-predicted bacterial phenotypic traits. (d) Stacked bar plot of BugBase-predicted bacterial phenotypic traits. (e) Clustered heatmap of FAPROTAX-predicted ecological functions. (f) Stacked bar plot of FAPROTAX-predicted ecological functions. CK, ultrapure water control; Hoagland, standard Hoagland nutrient solution; 5%, 10%, 15%, and 20%, swine biogas slurry treatments at corresponding v/v concentrations. All functional profiles were inferred from 16S rRNA gene amplicon data and should be interpreted as predicted functional potential rather than direct measurements of gene

abundance, gene expression, enzyme activity, or microbial metabolic activity.

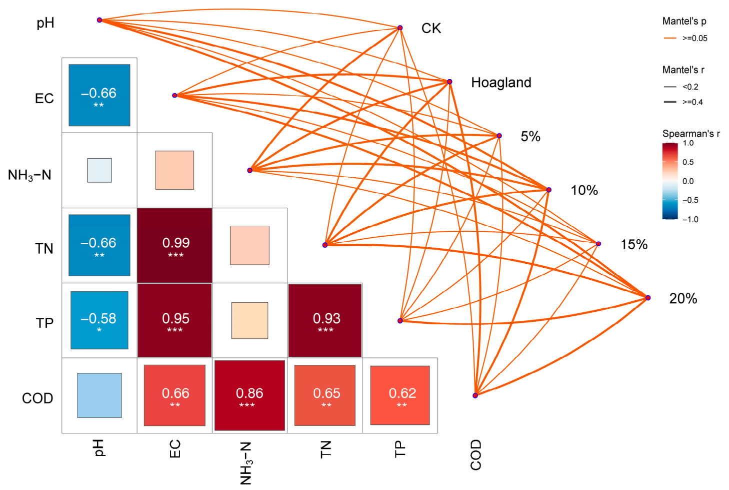

Figure S5. Mantel-test-based associations between environmental variables and bacterial community dissimilarities.

Note: Mantel test heatmap showing associations between physicochemical variables and bacterial community dissimilarities at the genus level. Bray–Curtis distance was used for the bacterial community matrix, and Euclidean distance was used for the environmental factor matrix. Environmental variables included pH, electrical conductivity (EC), ammonium nitrogen (NH_3-N), total nitrogen (TN), total phosphorus (TP), and chemical oxygen demand (COD). Line color and width indicate Mantel's r and statistical significance, respectively. The lower triangular heatmap shows Spearman correlations among environmental variables. CK, ultrapure water control; Hoagland, standard Hoagland nutrient solution; 5%, 10%, 15%, and 20%, swine biogas slurry treatments at corresponding v/v concentrations.

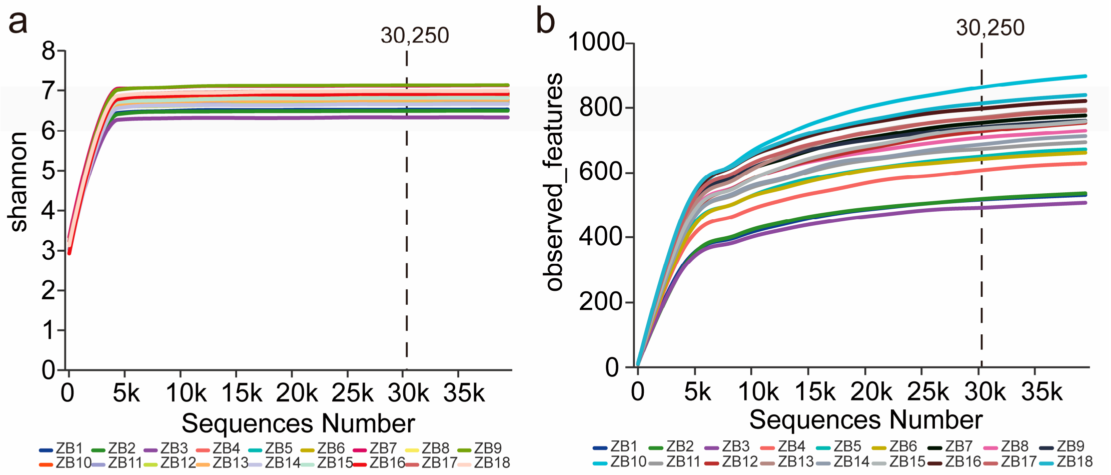

Figure S6. Rarefaction analysis of the 16S rRNA gene sequencing data for the 18 samples (ZB1–ZB18).

Note: (a) Rarefaction curves based on the Shannon diversity index. (b) Rarefaction curves based on observed features. The vertical dashed line indicates the rarefaction cutoff depth (30,250 sequences). In both analyses, the curves approached a plateau as sequencing depth increased, indicating that the selected cutoff was appropriate and that sequencing depth was sufficient to provide stable estimates of within-

sample bacterial diversity.
